# Comparative and Evolutionary Analysis of the Interleukin 17 Gene Family in Invertebrates

**DOI:** 10.1371/journal.pone.0132802

**Published:** 2015-07-28

**Authors:** Xian-De Huang, Hua Zhang, Mao-Xian He

**Affiliations:** 1 Key Laboratory of Tropical Marine Bio-Resources and Ecology, South China Sea Institute of Oceanology, Chinese Academy of Sciences, Guangzhou, China; 2 Guangdong Provincial Key Laboratory of Applied Marine Biology (LAMB), South China Sea Institute of Oceanology, Chinese Academy of Sciences, Guangzhou, China; Laboratoire de Biologie du Développement de Villefranche-sur-Mer, FRANCE

## Abstract

Interleukin 17 (IL-17) is an important pro-inflammatory cytokine and plays critical roles in the immune response to pathogens and in the pathogenesis of inflammatory and autoimmune diseases. Despite its important functions, the origin and evolution of IL-17 in animal phyla have not been characterized. As determined in this study, the distribution of the IL-17 family among 10 invertebrate species and 7 vertebrate species suggests that the IL-17 gene may have originated from Nematoda but is absent from *Saccoglossus kowalevskii* (Hemichordata) and Insecta. Moreover, the gene number, protein length and domain number of IL-17 differ widely. A comparison of IL-17-containing domains and conserved motifs indicated somewhat low amino acid sequence similarity but high conservation at the motif level, although some motifs were lost in certain species. The third disulfide bond for the cystine knot fold is formed by two cysteine residues in invertebrates, but these have been replaced by two serine residues in Chordata and vertebrates. One third of invertebrate IL-17 proteins were found to have no predicted signal peptide. Furthermore, an analysis of phylogenetic trees and exon–intron structures indicated that the IL-17 family lacks conservation and displays high divergence. These results suggest that invertebrate IL-17 proteins have undergone complex differentiation and that their members may have developed novel functions during evolution.

## Introduction

Interleukin 17 (IL-17) is an important pro-inflammatory cytokine and is a critical component of the immune response to pathogens and in the pathogenesis of inflammatory and autoimmune diseases [1–3]. IL-17 was initially identified as a cytokine secreted by T helper 17 (TH17) cells as one of its signature cytokines, and recent findings have indicated that IL-17 is also produced by other cell types, particularly by the innate immune cell populations involved in the inflammatory process [4]. IL-17 was first cloned and identified as cytotoxic T-lymphocyte (CTL)-associated antigen 8 (CTLA-8), a T-cell-derived cytokine with 58% identity to predicted open reading frame 13, HSVS13, of the T-lymphotropic *Herpesvirus saimiri* (known as virus IL-17) [5, 6]. Six IL-17 family members, IL-17A (the original IL-17), IL-17B, IL-17C, IL-17D, IL-17E (also known as IL-25) and IL-17F, have since been identified, and these proteins range in size from 20 to 30 kDa [7]. Among these family members, IL-17A and IL-17F share the highest amino acid sequence identity (50%), whereas IL-17E is the most divergent, showing 16% identity with IL-17A. Moreover, a novel type of IL-17 family gene (IL-17N) has recently been identified in teleosts [8]. Amino acid similarity among the family members is higher in the C terminus and in five spatially conserved cysteine residues, four of which form a cystine knot fold that forms two intrachain disulfide bonds. This cystine knot fold is similar to the canonical cystine knot observed in growth factors such as transforming growth factor (TGF)-β, endocrine glycoprotein hormones (e.g. chorionic gonadotrophin), platelet-derived growth factors (PDGFs), nerve growth factor (NGF) and other neurotrophins with six cysteines rather than four [9, 10].

Among the IL-17 family members, IL-17A and IL-17F are the best characterized, followed by IL-17C and IL-17E, while IL-17B and IL-17D have remained understudied [1]. Mechanistically, the biologically active form of IL-17 is a 35-kDa homodimer or heterodimer whose activity is dependent on the single-pass transmembrane receptors, IL-17 receptors (IL-17Rs), which have several conserved structural features, including an extracellular fibronectin III-like domain and a cytoplasmic SEF (similar expression to FGF)/IL-17R (SEFIR) domain. The IL-17Rs, as well as the cognate IL-17 family, have little homology with any other known receptors or ligands and therefore are thought to represent a distinct ligand–receptor signaling system that is highly conserved across vertebrate evolution. However, the exact mechanisms of IL-17 signaling have not been fully elucidated [1, 11].

Despite an accumulation of knowledge of the functions of IL-17 and their regulatory pathways, the number of pathways involving the IL-17 family remains unclear [1, 2, 12]. Some members of the IL-17 family are highly conserved among vertebrate organisms, but evolutionary analysis of the family has mainly been limited to vertebrates and a handful of invertebrates [8, 13], and little is known about its origin and evolution in animal phyla. For example, given that homology among IL-17 family members is only 16–50%, perhaps the IL-17A-like genes in some phyla may be too dissimilar to be identified but, interestingly, IL-17D has shown some degree of homology with IL-17-like proteins in primitive phyla such as worms [4]. The identification of similarities and differences in the IL-17 family among animal phyla, particularly invertebrates, could facilitate the elucidation of the functional evolution of this family, as well as allowing further functional verification. The recent large-scale sequencing of the transcriptomes and genomes of invertebrate species [14], particularly non-model organisms [15–17], represents a global survey that can be used to investigate IL-17 family members. For instance, in the purple sea urchin, about thirty IL-17 genes and two receptor genes were identified. Many of the ligands are linked in tandem arrays [18]. In this study, we determined the distribution of the IL-17 family among invertebrates, analyzed their exon–intron structures and phylogenetic trees, and explored their origin and evolutionary history in animal phyla.

## Materials and Methods

### Ethics statement

No specific permits were required for the field studies described, and the field studies did not involve endangered or protected species.

### Databases

The databases used in this study were obtained primarily from the National Center for Biotechnology Information (NCBI) Assembled RefSeq Genomes (http://www.ncbi.nlm.nih.gov/mapview/) and the DOE Joint Genome Institute (JGI) (http://genome.jgi.doe.gov/) websites. *Nematostella vectensis* (Cnidaria), *Caenorhabditis briggsae* (Nematoda), *Capitella teleta* (Annelida), *Helobdella robusta* (Annelida), *Lottia gigantea* (Mollusca), *Daphnia pulex* (Arthropoda), *Trichoplax adhaerens* (Placozoa) and *Branchiostoma floridae* (Chordata) from JGI and *Amphimedon queenslandica* (Porifera/Spongia), *Hydra magnipapillata* (Cnidaria), *Caenorhabditis elegans* (Nematoda), *Saccoglossus kowalevskii* (Hemichordata), *Acyrthosiphon pisum* (Insecta of Arthropoda, same as below), *Apis mellifera* (Insecta), *Drosophila melanogaster* (Insecta), *Ciona intestinalis* (Chordata) and Protozoa from NCBI Assembled RefSeq Genomes were individually analyzed by BLASTP. For *Strongylocentrotus purpuratus* (Echinodermata), BLASTP was performed utilizing both the NCBI Assembled RefSeq Genomes and Sea Urchin Genome Database (http://www.spbase.org/SpBase/) datasets. The protein data for *Crassostrea gigas* (Mollusca) were downloaded from NCBI, and a local BLAST protein database was constructed for the BLAST search. For *Pinctada fucata* (Mollusca), BLASTP was run on *Pinctada fucata* Genome Ver. 1.00 (http://marinegenomics.oist.jp/genomes/ncbiblast/search?project_id=20). BLASTP analysis was conducted on vertebrate taxa including *Danio rerio*, *Oryzias latipes*, *Gallus gallus*, *Homo sapiens*, and *Xenopus (Silurana) tropicalis* from NCBI Assembled RefSeq Genomes. For *Takifugu rubripes*, sequences reported by Hiroki Korenaga et al. [8] were used.

### Identification of IL-17 genes

BLAST searching methods were used to identify IL-17 proteins. The amino acid sequences of the IL-17 domain previously identified in *P*. *fucata* IL-17 (JX971444) and *C*. *gigas* IL-17 (ABO93467) were used as query sequences to BLAST against the protein database of each genome for the species mentioned above [19, 20]. The threshold E-value was set to range from 3 to 10 with 50 maximum target sequences, to identify a maximal number of candidate sequences, and other parameters were left at the default values. After the corresponding hits were downloaded from the BLAST results, the sequences were examined using the NCBI CDS program (Batch CD-search, http://www.ncbi.nlm.nih.gov/Structure/bwrpsb/bwrpsb.cgi) with default cutoff parameters to remove sequences that did not contain the IL-17 domain. For the maximum target sequences obtained, the hit sequence with the maximum numeric E-value was used as the query sequence to BLAST against the protein database of the corresponding species. The sequences from the genome of each species were analyzed independently using Clustal Omega Multiple Sequence Alignment (http://www.ebi.ac.uk/Tools/msa/clustalo/) to eliminate redundant sequences. To simplify the presentation and subsequent discussion, the longest isoform sequence was retained, and other isoforms were removed. For incomplete sequences containing the complete IL-17 domain, only the longest sequence was retained.

### Sequence analysis and amino acid alignment

Batch CD-search was used to analyze the domain among the IL-17 protein sequences identified, and MEME 4.9.1 (Motif-based sequence analysis tools, http://meme.nbcr.net/meme/) was used to identify motifs in the IL-17 protein sequences. Signal peptides were predicted using SignalP 4.1 [21]. Comparison and phylogenetic analysis were performed using Clustal Omega multiple sequence alignment and the MEGA 6.06 software using neighbor-joining (NJ) methods and performing 10,000 bootstrap replications [22].

### Exon-intron structure and location of IL-17 genes

For the IL-17 amino acid sequences, the corresponding nuclear sequences, including the EST and genomic sequences, were obtained. Spidey, an mRNA-to-genomic alignment program (http://www.ncbi.nlm.nih.gov/spidey/), was used to analyze exon-intron structures. Owing to the use of draft genomes, some IL-17 exon–intron structures were not available. Meanwhile, the genomic location of IL-17 genes were analyzed, using the NCBI mapview browsers.

## Results

### Genome-wide identification of IL-17 genes from invertebrates

By performing BLAST searches of databases encompassing a wide spectrum of organisms from Protozoa to *B*. *floridae*, a total of 54 putative IL-17 genes were identified from 10 invertebrate genomes: *C*. *briggsae* (1) (Nematoda), *C*. *elegans* (2) (Nematoda), *C*. *teleta* (6) (Annelida), *L*. *gigantean* (6) (Mollusca), *C*. *gigas* (7) (Mollusca), *P*. *fucata* (12) (Mollusca), *D*. *pulex* (1) (Arthropoda), *S*. *purpuratus* (6) (Echinodermata), *C*. *intestinalis* (5) (Chordata) and *B*. *floridae* (8) (Chordata). Detailed information on the IL-17 genes identified from each genome surveyed is listed in Table 1 and the protein sequences in S1 Dataset. IL-17 homologs could not be identified in Porifera (*A*. *queenslandica*), Cnidaria (*H*. *magnipapillata* and *N*. *vectensis*), Placozoa (*T*. *adhaerens*), Hemichordata (*S*. *kowalevskii*), Insecta (such as *A*. *pisum*, *A*. *mellifera* and *D*. *melanogaster*), and Protozoa. For comparison, IL-17 homologs from vertebrates including *D*. *rerio* (5), *T*. *rubripes* (6), *Oryzias latipes* (6), *G*. *gallus* (4), *H*. *sapiens* (6) and *X*. *tropicalis* (7) are also listed in Table 1. These results provide a concise picture of the IL-17 gene distribution, and reveal that the IL-17 gene arose not in a lower invertebrate such as Porifera and Cnidaria but in Nematoda species such as *C*. *briggsae* and *C*. *elegans*; it subsequently emerged in some mollusks. Not all arthropods exhibit IL-17 gene loss; *D*. *pulex* contains a sequence homologous to IL-17. However, no IL-17 homologous sequence has been identified in other arthropods. These results suggest that IL-17 genes may have originated from Nematoda.

**Table 1:** Summary of IL-17 genes.

Classes	Species and putative genes name	Length	Location of CDS	E-Value	Signal peptide	Genomic location	Number of intron
Nematoda	C.briggsae XP_002637129.1 protein CBG09631	145	48–126	0.000000472	/	chromosome V	0
	C.elegans NP_505700.2 Protein F25D1.3	189	92–170	3.21E-08	Yes	chromosome V	2
	C.elegans NP_510131.2 Protein T22H6.1	221	122–195	0.00502391	NO	chromosome X	6
Annelida	C.teleta 199819	233	150–226	2.19E-08	NO	scaffold_169	5
	C.teleta 198235	366	286–360	0.000000032	Yes	scaffold_451	1
	C.teleta 206957	202	112–195	4.51E-09	Yes	scaffold_22	1
	C.teleta 216301	180	91–169	0.000216745	Yes	scaffold_314	0
	C.teleta 205055	166	81–157	0.0000268	NO	scaffold_79	1
	C.teleta 209749	348	260–338	0.000489314	NO	scaffold_52	4
			237–278	0.00378139^a^			
Mollusca	L.gigantea 152638	187	97–173	0.000000148	Yes	**scaffold_2**	0
	L.gigantea 169526	191	98–174	0.00246412	Yes	scaffold_86	1
	L.gigantea 164174	180	91–179	0.00000767	Yes	scaffold_44	2
	L.gigantea 228210	191	105–183	2.85E-11	Yes	**scaffold_2**	0
	L.gigantea 172928	154	71–144	0.000000264	Yes	scaffold_144	0
	L.gigantea 159302	186	94–169	0.00000077	Yes	scaffold_20	1
	C.gigas ABO93467.1 IL-17	200	96–179	3.88E-13	Yes		/*
	C.gigas EKC33705.1 protein CGI_10020734	190	100–170	9.85E-09	Yes	scaffold_698	2
	C.gigas EKC26195.1 protein CGI_10027182	167	81–161	2.1E-10	Yes	scaffold_1599	2
	C.gigas EKC33786.1 protein CGI_10014828	141	67–131	0.000172218	NO	scaffold_689	1
	C.gigas EKC38792.1 protein CGI_10026592	132	46–124	0.000000893	NO	scaffold_313	0
	C.gigas EKC33462.1 proteinGI_10015251	148	73–144	2.97E-11	Yes	scaffold_723	1
	C.gigas EKC32654.1 protein CGI_10004922	132	53–128	0.000525737	Yes	scaffold_806	0
	P.fucata 1712.1_51392.t1	208	121–196	1.06E-13	NO	**scaffold_1712.1**	1
	P.fucata 1712.1_51391.t1	158	62–137	0.0000021	NO	**scaffold_1712.1**	2
	P.fucata 1712.1_51394.t1	169	90–160	0.00000792	/	**scaffold_1712.1**	0
	P.fucata 20923.1_18751.t1	261	29–91	0.000000303	/	scaffold_20923.1	1
	P.fucata 24776.1_26199.t1	165	71–152	6E-16	/	scaffold_24776.1	0
	P.fucata 27731.1_19195.t1	189	96–170	4.92E-10	/	scaffold_27731.1	1
	P.fucata 27889.1_19207.t1	165	76–151	0.00161047	NO	scaffold_27889.1	1
	P.fucata 32457.1_48078.t1	146	65–138	9.08E-11	NO	scaffold_32457.1	0
	P.fucata 204780.1_72074.t1	145	64–137	0.000000303	Yes	scaffold_204780.1	0
	P.fucata 8564.1_24423.t1	96	2–75	7.74E-08	NO	**scaffold_0_8564.1**	0
	P.fucata 8564.1_24422.t1	206	103–174	0.0000643	NO	**scaffold_0_8564.1**	0
	P.fucata JX971444.1 IL-17	194	91–171	0.00000608	Yes	scaffold_9999.1	1
Arthropoda	D.pulex 125692	233	156–225	0.00858264	NO	scaffold_13069	0
Echinodermata	S.purpuratus SPU_019350.1	537	454–532	5.37E-16	Yes	**scaffold_1105**	2
			279–351	2.25E-13			
			104–176	2.9E-12			
	S.purpuratus SPU_022838.1	379	272–354	5.68E-16	NO	scaffold_1325	4
			110–181	4.18E-11			
	S.purpuratus SPU_030196.1	204	113–194	1.02E-14	Yes	**scaffold_2038**	2
	S.purpuratus SPU_030199.1	236	153–232	5.26E-13	/	**scaffold_1105**	1
	S.purpuratus SPU_030204.1	215	122–209	7.92E-10	NO	scaffold_2240	2
	S.purpuratus SPU_030198.1	344	256–338	1.46E-10	Yes	**scaffold_2038**	2
Chordata	C.intestinalis XP_004227512.1 IL-17D-like	179	96–173	8.57E-14	Yes	unplaced scaffold	4
	C.intestinalis NP_001123348.1 IL-17-3	186	103–180	1.72E-12	NO	**chromosome 1**	2
	C.intestinalis NP_001123346.1 IL-17-2	171	92–169	5.15E-30	Yes	**chromosome 1**	2
	C.intestinalis NP_001123347.1 IL-17-1	171	86–164	8.46E-31	Yes	**chromosome 1**	2
	C.intestinalis 203738	199	117–196	1.03E-12	Yes	**chromosome 1**	2
	B.floridae 91950	434	347–428	1.18E-24	NO	scaffold_216	0
	B.floridae 117645	177	93–174	0.00000461	Yes	scaffold_6	1
	B.floridae 230778	93	3–84	8.51E-27	NO	**scaffold_275**	1
	B.floridae 127768	199	114–194	9.77E-26	Yes	**scaffold_275**	3
	B.floridae 92872	177	93–171	5.24E-10	Yes	scaffold_229	1
	B.floridae 132638	470	326–435	6.28E-49^b^	Yes	scaffold_746	9
			99–173	5.31E-14^c^			
			438–467	1.89E-09			
	B.floridae 94821	254	169–248	0.00000556	Yes	scaffold_258	2
	B.floridae 66165	151	69–144	0.000114888	Yes	scaffold_9	1
Vertebrate	D.rerio XM_002666436.1 protein LOC100329556	126	52–125	0.000000225	Yes	chromosome 23	2
	D.rerio NP_001018634.1 IL-17a/f2	140	58–136	4.14E-21	Yes	**chromosome 17**	2
	D.rerio NP_001018625.1 IL-17D	212	96–178	4.35E-40	Yes	chromosome 9	1
	D.rerio NP_001018623.1 IL-17a/f1	153	67–147	3.4E-35	Yes	**chromosome 17**	2
	D.rerio NP_001018626.1 IL-17a/f3	162	73–155	4.1E-33	Yes	chromosome 20	2
	T.rubripes BAI82582.2 IL-17C-2	160	73–154	6.57E-24	Yes	**chromosome 13**	2
	T.rubripes BAI82581.2 IL-17C-1	161	81–156	5.99E-17	Yes	scaffold_430	2
	T.rubripes BAI82580.1 IL-17A/F-3	158	58–151	3.32E-21	Yes	chromosome 16	3
	T.rubripes BAI82579.1 IL-17A/F-2	144	58–138	2.01E-19	Yes	**chromosome 13**	2
	T.rubripes BAI82578.1 IL-17A/F-1	160	72–153	3.17E-24	Yes	**chromosome 13**	2
	T.rubripes BAI82584.1 IL-17N	139	55–133	8.57E-13	Yes	scaffold_264	2
	O.latipes NP_001191715.1 IL-17A/F-3	157	60–150	8.62E-22	Yes	chromosome 24	3
	O.latipes NP_001191713.1 IL-17A/F-2	142	56–135	5.08E-22	Yes	**ultracontig 46**	2
	O.latipes NP_001191714.1 IL-17A/F-1	152	66–144	3.49E-24	Yes	**ultracontig 46**	2
	O.latipes NP_001191716.1 IL-17D	211	97–179	1.29E-36	Yes	chromosome 21	1
	O.latipes NP_001191723.1 IL-17C	165	72–162	1.08E-16	Yes	chromosome 6	2
	O.latipes NP_001191717.1 IL-17N	139	55–134	2.65E-12	Yes	chromosome 9	2
	G.gallus XP_003641993.2 IL-17C	188	97–183	3.87E-33	Yes	chromosome 11	2
	G.gallus XP_426223.4 IL-17F	169	82–160	1.94E-33	Yes	**chromosome 3**	2
	G.gallus NP_989791.1 IL-17F precursor	169	82–162	1.68E-34	Yes	**chromosome 3**	2
	G.gallus XP_004944893.1 IL-17B isoform X4	243	159–243	4.62E-24	Yes	chromosome 13	2
	X.tropicalis NP_001107719.1 IL-17D	204	89–170	1.05E-42	Yes	scaffold_2	2
	X.tropicalis XP_004915038.1 IL-17A-like	160	61–140	3.29E-32	Yes	**scaffold_5b**	2
	X.tropicalis XP_002942041.2 IL-17C	198	111–194	9E-35	Yes	scaffold_4	2
	X.tropicalis XP_002932904.1 IL-17D-like	156	73–153	7.37E-23	Yes	scaffold_10	2
	X.tropicalis XP_004915036.1 IL-17F	185	97–177	5.61E-33	NO	**scaffold_5b**	2
	X.tropicalis NP_001006699.1 IL-17B	203	115–203	1.8E-40	Yes	scaffold_3	2
	X.tropicalis XP_004915037.1 IL-17A-like	149	62–140	3.71E-30	Yes	**scaffold_5b**	2
	H.sapiens NP_612141.1 IL-17D	202	89–171	9.32E-45	Yes	chromosome 13	2
	H.sapiens NP_443104.1 IL-17F	163	76–156	6.29E-40	Yes	**chromosome 6**	2
	H.sapiens NP_055258.1 IL-17B	180	95–180	1.16E-33	Yes	chromosome 5	2
	H.sapiens NP_002181.1 IL-17A	155	68–148	9.03E-43	Yes	**chromosome 6**	2
	H.sapiens NP_037410.1 IL-17C	197	103–193	1.4E-37	Yes	chromosome 16	2
	H.sapiens NP_073626.1 IL-25 isoform 1	177	84–172	4.18E-26	Yes	chromosome 14	1

Syntenic loci in the species was indicated in bold

The extra domains besides IL-17 domain:

^a.^ YccV-like domain;

^b.^ RPA2b aaRSs OBFlike domain;

^c.^ classII aaRS like core domain

Furthermore, this table indicates that the length of invertebrate IL-17 proteins generally ranges from 100 to 250 amino acids but fluctuates greatly when compared with vertebrate homologs. More drastic changes are found at the EST or exon sequence level, ranging from a few hundred to about two thousand base pairs, although the sequence data of some species mentioned above are insufficient or contain errors. The IL-17 domains generally contain approximately 70 amino acids and are located in the C-terminal region of the sequences. Interestingly, there are some exceptions: 1) three IL-17 superfamily domains with repetitive protein sequences in *S*. *purpuratus* SPU_019350.1; 2) two IL-17 superfamily domains with different protein sequences in *S*. *purpuratus* SPU_022838.1; 3) one IL-17 domain that partially overlaps with an incomplete YccV-like superfamily domain in *C*. *teleta* 209749; and 4) multi-domains with the N-terminal anticodon recognition domain of lysyl-tRNA synthetases (LysRS_N), the IL-17 superfamily, incomplete lysyl-tRNA synthetases, and the Class II tRNA amino-acyl synthetase-like catalytic core domain (LysRS_core) in *B*. *floridae* 132638. In addition, some IL-17 proteins, including *C*. *elegans* protein C44B12.6, isoform a (CDH93392.1), *P*. *fucata* 8548.1_09780.t1 and *S*. *purpuratus* SPU_030197.1, contain incomplete IL-17 domains and are listed in S1 Dataset but not Table 1. These results suggest that IL-17 protein sequences have undergone rapid and continual changes which may have led to a change in their function.

### Conserved residues and motifs in IL-17 proteins

**Fig 1:**
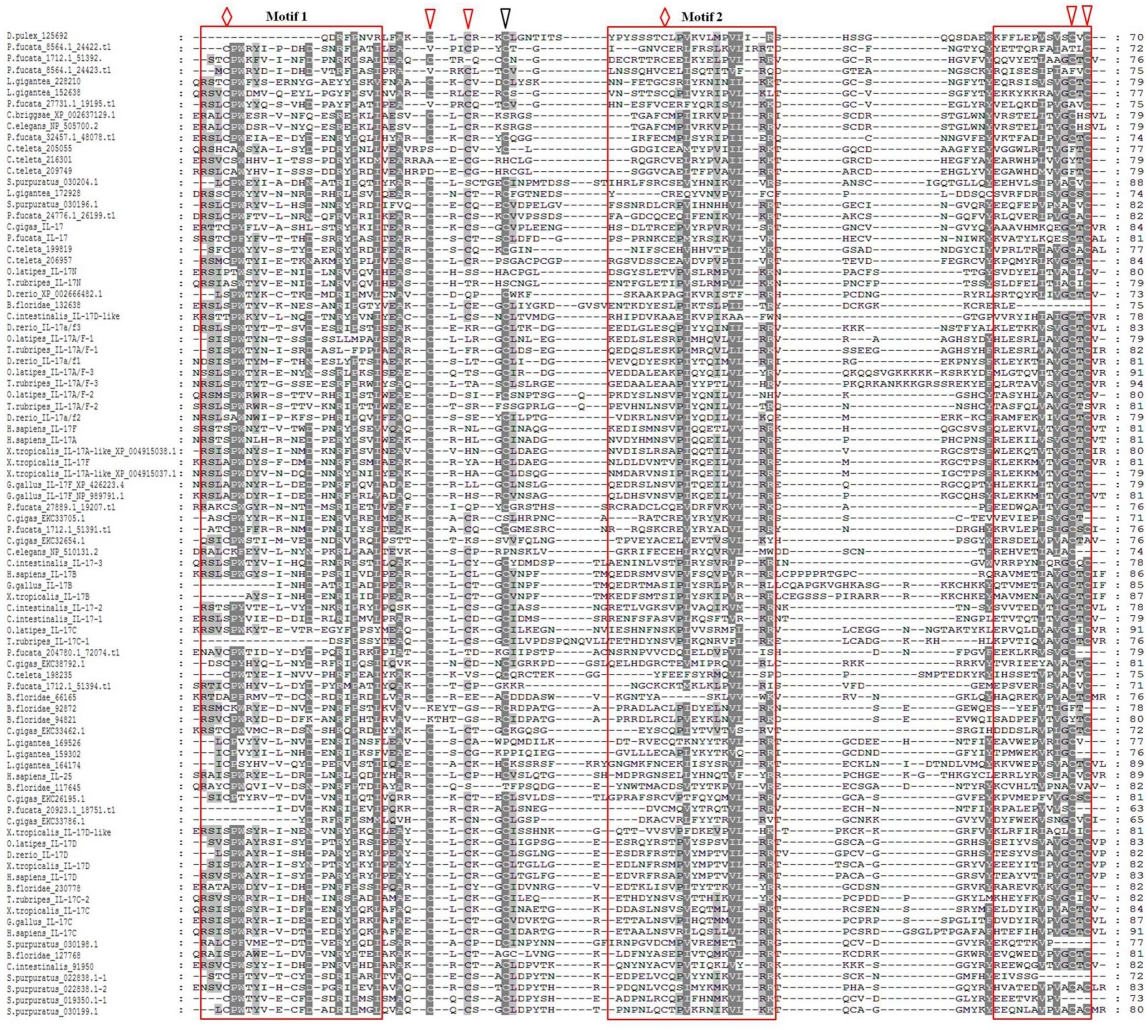
Clustal Omega amino acid sequence alignment and three conserved motifs of 89 IL-17 domains. The shading of the alignment represents different degrees of conservation among sequences. The dark shading indicates identical residues. Arrows indicate the positions of cysteine residues. A rhombus indicates the positions in which some cysteine residues have been replaced by serine residues.

To clarify the relationships among IL-17 proteins from different species, multiple alignment analysis of the IL-17 domains was performed using Clustal Omega. The results indicated that the distribution of amino acid residues is not conserved in IL-17 domains, as illustrated in Fig 1, or in full-length invertebrate IL-17 proteins (data not shown). However, five cysteine residues (marked with arrows) were basically conserved, four (red arrows) of which are important for the cystine knot fold. Remarkably, there is a third disulfide bond for the cystine knot fold that is formed by the two cysteine residues in invertebrates, except for Chordata (*B*. *floridae* and *C*. *intestinalis*), in which the cysteine residues have been replaced by two serine residues (red rhombus).

**Fig 2:**
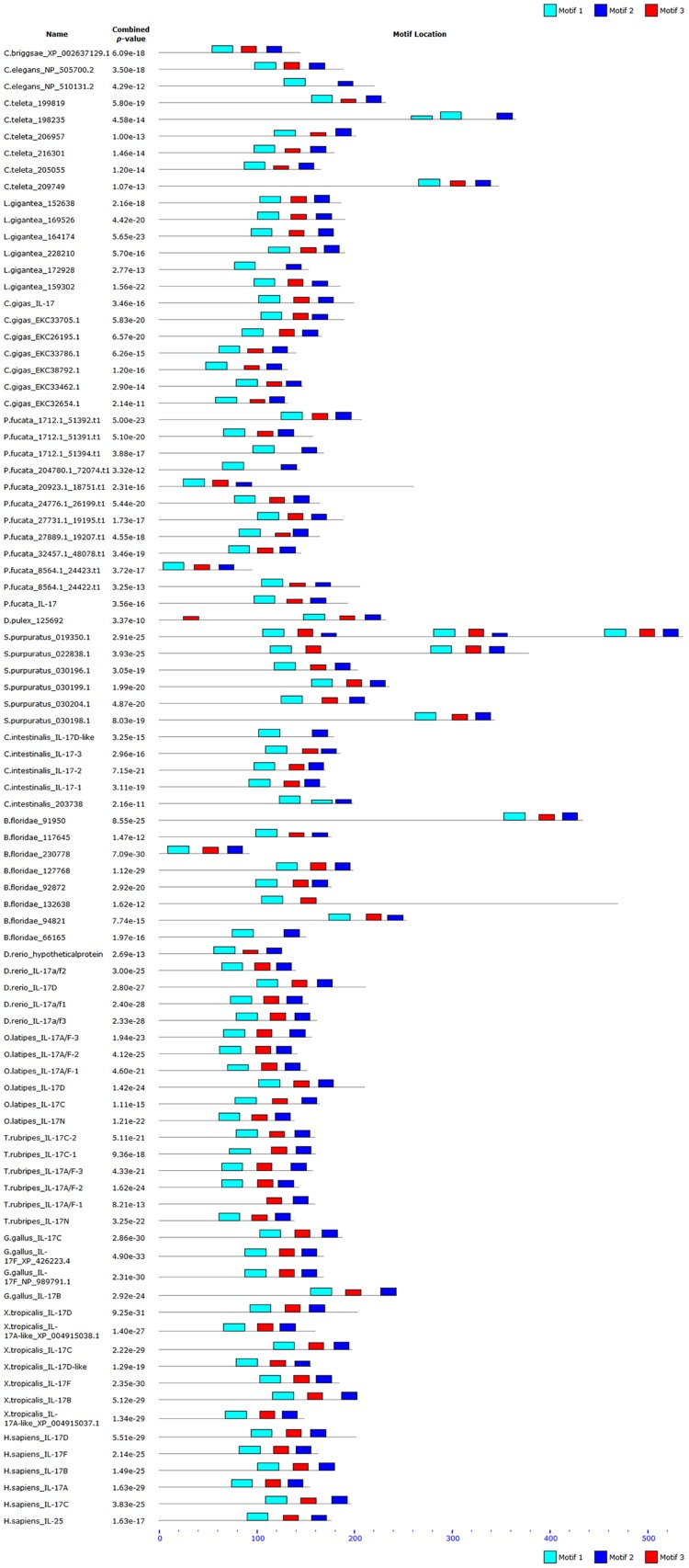
Combined block diagrams of three conserved motifs in IL-17 proteins. The motifs in IL-17 protein sequences were analyzed by MEME 4.9.1. Non-overlapping sites are indicated by a *p*-value greater than 0.0001. The height of the motif “block” is proportional to the–log (*p*-value), truncated at the height of a motif with a *p*-value of 1*e* ^–10^.

MEME was performed to discover conserved motifs within the IL-17 proteins and IL-17 domains. The sequences of the motifs in IL-17 domains are presented in Fig 2 and the combined motif block diagrams are shown in S1 Fig. From Fig 2, it can be observed that all 89 predicted IL-17 protein sequences contain the following three motifs: motif 1 (xY[VR]I[ND]xDPNR[IYF]Pxx[IL]xEA[RK]CL), motif 2 (YExxxEx[VI][APT]V[GA]CTC[VA]) and motif 3 (LN[SC]VP[IV]YQxILVLR[RK]). Similar motifs (including sequence logos) were observed in the IL-17 domains (S1B Fig); only the motif name is different, when compared with that of the full-length IL-17 proteins. Furthermore, as shown in Fig 2, motif 1 was only absent from *T*. *rubripes* IL-17A/F-1, and motif 2 was absent from the N-terminus of the IL-17 domain in *S*. *purpuratus* SPU_022838.1 and *B*. *floridae* 132638. Motif 3 was absent from *C*. *elegans* NP_510131.2, *C*. *teleta* 192928, *P*. *fucata* 1712.1_51394.t1 and 204780.1_72074.t1, *C*. *intestinalis* IL-17D-like and 203738, and *B*. *floridae* 66165. In addition, a comparison of the motifs in IL-17 proteins and IL-17 domains indicated that these motifs were primarily located in IL-17 domains, suggesting that, although the amino acid sequence identity of IL-17 proteins is rather low, they exhibit greater conservation at the motif level.

Meanwhile, SignalP was performed to predict signal peptides at the N-terminal IL-17 proteins. As shown in Table 1, in vertebrates, 33 out of 34 IL-17 proteins had a predicted signal peptide, except for *X*. *tropicalis* IL-17F. In contrast 32 out of 54 of invertebrate IL-17 proteins had the predicted signal peptide, while 1/3 (18 out of 54 IL-17 proteins) had no signal peptide, and 6 IL-17 proteins were unknown due to their incomplete protein sequences. The results indicated that many of IL-17 proteins in invertebrates have no predicted signal peptide, suggesting that they might be not be secreted proteins.

### Phylogenetic analysis and classification of invertebrate IL-17 proteins

**Fig 3:**
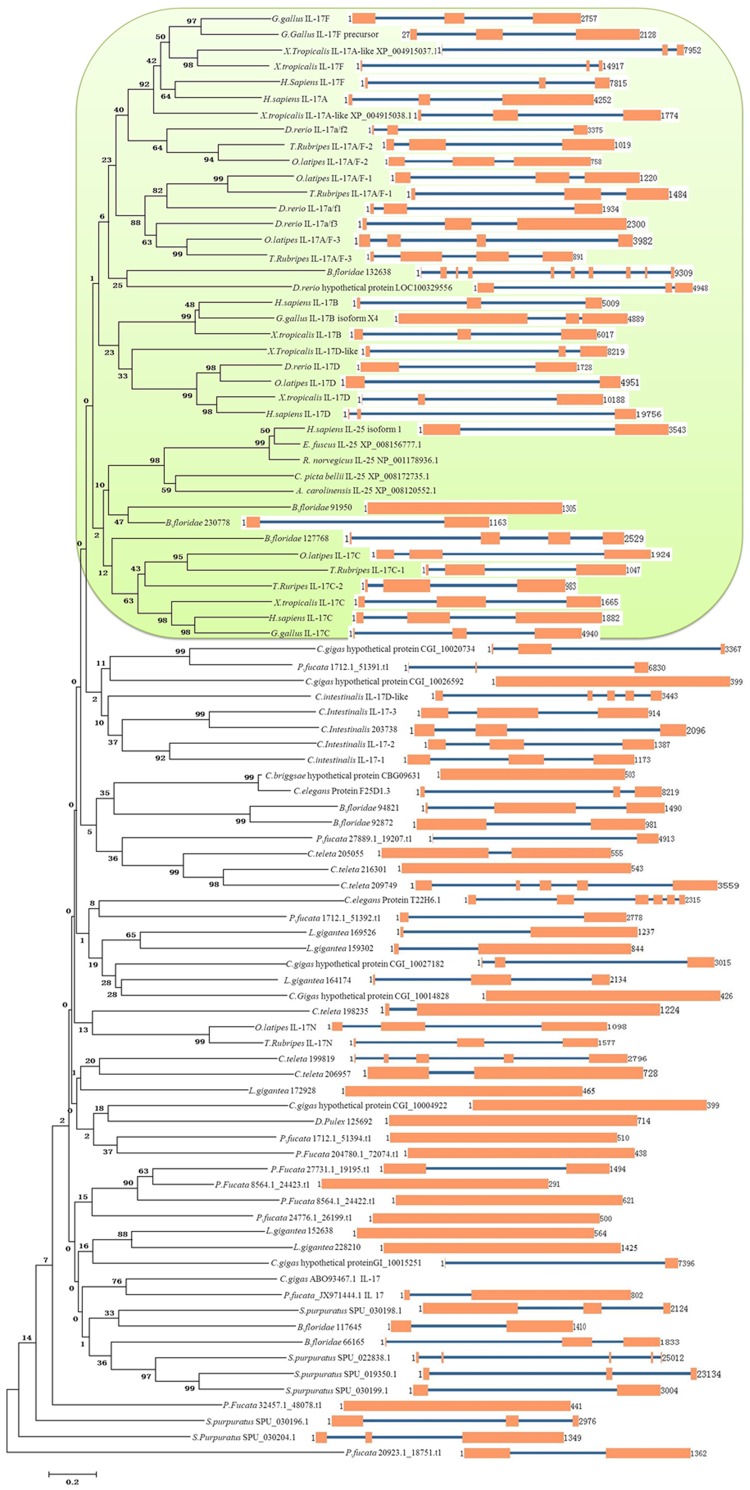
Phylogenetic and gene structure analysis of the IL-17 gene. The phylogenetic tree was constructed using the neighbor-joining method in the MEGA 6.06 software. Each node is represented by a number that indicates the bootstrap value for 10,000 replicates. The scale bar represents 0.2 substitutions per sequence position (left). The right side illustrates the exon–intron organization of the corresponding IL-17 genes. The exons and introns are represented by orange boxes and blue lines, respectively. The numbers indicate the length of the gene. The extra IL-17 protein sequences (*Rattus norvegicus* IL-25 (NP_001178936.1), *Chrysemys pictabellii* IL-25 (XP_008172735.1), *Alligator sinensis* IL-25 (XP_008120552.1)) have not been listed in S1 Dataset.

To investigate the potential evolutionary relationships of the IL-17 family, phylogenetic trees were constructed based on the amino acid sequences of the full-length proteins. The phylogenetic tree based on full-length sequences in the NJ analysis was divided into many subgroups (Fig 3). Nearly all vertebrate IL-17 proteins were located in one subgroup (the light green area), in agreement with the phylogenetic tree of vertebrate IL-17 proteins presented in S2 Fig. In addition, many of the invertebrate IL-17 proteins form a large group subsequently divided into several subgroups. In general, the IL-17 proteins from a single species were distributed over different groups. These results indicate that, during evolution, invertebrate IL-17 proteins underwent complex differentiation and include far more than the 7 members (IL-17A-F and IL-17N) found in vertebrates, suggesting that these IL-17 proteins may have developed novel functions during evolution.

### Exon-intron structure and location of IL-17genes

The exon-intron structure of IL-17 genes in invertebrates and vertebrates was examined to obtain further insight into the possible structural evolution of these genes. As shown in Table 1 and Fig 3, in vertebrates, 29 out of 34 IL-17 genes had two introns, while three members contained only one intron and two members had three introns. By contrast, in invertebrates, the intron number of IL-17 was more variable, but generally (49 of 54) ranged from 0 to 3. The exceptions were genes with 4 introns (*C*. *teleta* 209749, *C*. *intestinalis* IL-17D-like, *S*. *purpuratus* SPU_022838.1), 5 introns (*C*.*teleta* 199819), 6 introns (*C*. *elegans* NP_510131.2), 9 introns (*B*. *floridae* 132638), and *C*. *gigas* ABO93467.1, which had no corresponding genomic structure available because of the draft status of its genome. These results indicate that the number of IL-17 introns has fluctuated greatly in invertebrates but has been relatively stable in vertebrates, further indicating the complex evolution of IL-17 proteins.

Interestingly, many invertebrate IL-17 genes are found to be located in the same scaffold, including *L*. *gigantea* 152638 and 172928 in scaffold_2; *P*. *fucata* 1712.1_51392.t1, 1.0_1712.1_51391.t1 and 1712.1_51394.t1 in scaffold_1712.1, and 8564.1_24423.t1 and 8564.1_24422.t1 in scaffold_8564.1; *S*. *purpuratus* SPU_019350.1 and SPU_030199.1 in scaffold_1105, and SPU_030196.1 and SPU_030198.1 in scaffold_2038; *C*. *intestinalis* IL-17-3, IL-17-2, IL-17-1 and 203738 in chromosome 1; *B*. *floridae* 230778 and 127768 in scaffold_275. In contrast, many vertebrate IL-17 genes are on the same chromosome, such as *D*. *rerio* IL-17a/f2 and IL-17a/f1 on chromosome 17; *T*. *rubripes* IL-17C-2, IL-17A/F-1 and IL-17A/F-2 on chromosome 13; *O*. *latipes* IL-17A/F-2 and IL-17A/F-1 in ultracontig 46; *G*. *gallus* IL-17F and IL-17F precursor on chromosome 3; *X*. *tropicalis* IL-17A-like (XP_004915038.1), IL-17F, IL-17A-like (XP_004915037.1) in scaffold_5b; and *H*. *sapiens* IL-17F and IL-17A on chromosome 6. This result indicates that several IL-17 genes are present in tandem on the same chromosome and may have been derived from gene duplication.

## Discussion

As an important regulatory cytokine, IL-17 is involved in and mediates cell–cell communication for many biological processes, particularly host defense responses and inflammatory diseases [1, 2]. However, the functions and characteristics of the invertebrate IL-17 family have not been well characterized [13, 14, 19, 23]. The recent release of a number of invertebrate genome databases may provide new insights into the IL-17 family. In the present study, we identified and summarized 54 IL-17-encoding genes in invertebrates and compared them with 28 vertebrate homologs, to investigate their origin and diversification. IL-17 genes were identified in invertebrates including Nematoda (*C*. *briggsae* and *C*. *elegans*), Annelida (*C*. *teleta*), Mollusca (*L*. *gigantean*, *C*. *gigas* and *P*. *fucata*), Arthropoda (*D*. *pulex*), Echinodermata (*S*. *purpuratus*) and Chordata (*C*. *intestinalis* and *B*. *floridae*) but were absent from Porifera (*A*. *queenslandica*), Cnidaria (*N*. *vectensis* and *H*. *magnipapillata*), Hemichordata (*S*. *kowalevskii*), Placozoa (*T*. *adhaerens*) and Insecta (such as *A*. *pisum*, *A*. *mellifera*, and *D*. *melanogaster*), as well as Protozoa. The number of IL-17 genes in each species was highly variable, ranging from 1 (*C*. *briggsae*) to 12 (*P*. *fucata*), which may reflect their unusually high evolutionary rate (Table 1). While the absence of the cytokine IL-17 family, which functions in cell-cell communication, in Protozoa and simple, ancient lower invertebrates such as *A*. *queenslandica* and *H*. *magnipapillata* was not unanticipated, it is puzzling that IL-17 genes were missing from Hemichordata (*S*. *kowalevskii*) and relatively high insects. This result is partially supported by a report by Simakov et al. that, although mollusks and annelids are related to flies, nematodes and flatworms within the protostomes, the genome organization, gene structure and functional content of these species are in many ways more similar to those of invertebrate deuterostomes (such as amphioxus and sea urchin) [16]. These similarities include features of bilaterian and/or metazoan genomes that have been lost or diverged in many protostome genomes.

Furthermore, immune gene families are usually under more intense evolutionary pressure, and rapid evolutionary changes are frequently observed for effector proteins such as cytokine IL-17 [24, 25]. In this study, the length and domain number of some IL-17 proteins varied greatly, suggesting broadened or reduced functions. For example, *B*. *floridae* 132638 contains not only the IL-17 domain but also the LysRS_N and incomplete LysRS core domain. LysRS_N is a beta-barrel domain (OB fold) involved in binding the tRNA anticodon stem–loop. LysRS enzymes are homodimeric class 2b aminoacyl-tRNA synthetases (aaRSs), which catalyze the specific attachment of amino acids to their cognate tRNAs during protein biosynthesis [26]. IL-17 enhances the expression of multiple pro-inflammatory cytokines, particularly members of the CXC chemokine family, through mRNA stabilization via an AUUUA/Tristetraprolin-independent sequence [27, 28]. By contrast, some IL-17 proteins contain incomplete IL-17 domains (S1 Dataset). This study also demonstrated that, although the amino acid sequence similarities of the IL-17 proteins were rather low, the motifs were highly conserved, although some motifs were lost in certain species. Given that these conserved motifs are located, to a great extent, in IL-17 domains, they provide the base for IL-17 domains and proteins. Significantly, there is a third disulfide bond for the cystine knot fold in invertebrate IL-17 proteins, suggesting that they may possess the canonical disulfides of the cystine knot, which belongs to the canonical cystine knot fold superfamily, with members such as the NGF subfamily; This is until in Chordata (*B*. *floridae* and *C*. *intestinalis*), where the two cysteine residues have been replaced by the corresponding serine residues [29, 30]. Unlike almost all vertebrate IL-17 proteins, which contain a predicted signal peptide, a significant proportion of those of invertebrates have no predicted signal peptide. The secretory signal peptide targets its passenger protein for translocation across the endoplasmic reticulum membrane in eukaryotes and the cytoplasmic membrane in prokaryotes [31]. The invertebrate IL-17 proteins without a predicted signal peptide may perform a different function from that of their vertebrate counterparts. Furthermore, some IL-17 genes were found to exhibit conserved synteny, which reveals a close evolutionary relationship between two genes or even two species and suggests that they may be derived from a common ancestor. This may also partially explain why IL-17A-like genes in some phyla may be too dissimilar to be identified. These results suggest that IL-17 proteins and their functions have been continuously undergoing dynamic change through evolution.

Previous studies of genomic organization involving phylogenetic analysis have revealed that the genomic organization of the vertebrate IL-17 family has been basically conserved through evolution [8, 13]. In mammals, the IL-17 family is generally divided into six members (IL-17A–F) or subgroups, and IL-17N is also present in fish. Furthermore, each member of the IL-17 family has different functions, with the exception of IL-17A and IL-17F. In this study, phylogenetic analysis indicated that there are many subgroups of the IL-17 family in invertebrates that likely produce numerous IL-17 family members, far more than the 7 known members in vertebrates (IL-17A-F and IL-17N), which suggests that the invertebrate proteins have undergone high divergence, including in their function. Additionally, introns may affect gene expression by increasing the time required to transcribe the gene, and intron-containing and intronless versions of otherwise identical genes can exhibit dramatically different expression profiles [32, 33]. While there is no universal intron requirement for eukaryotic gene expression, in many cases transgene expression can be dramatically increased by the addition of just one generic intron to the cDNA [34, 35]. This may give a partial explanation for the change in the number of IL-17 introns from invertebrates to vertebrates. Although intron evolution is a dynamic process in eukaryotes [36], the comparison of IL-17 family gene organization revealed that the IL-17 family gene has not been very highly conserved throughout evolution. The more drastic changes in the exons also strengthen this observation. In general, from the perspective of both phylogenetics and genomic organization, the IL-17 family lacks conservation and exhibits high divergence, suggesting that invertebrate IL-17 proteins have undergone complex differentiation and that their members may have developed novel functions during evolution.

In the progression from unicellular protozoans to multicellular animals, the capability for more advanced and complicated communication and cooperation among cells was acquired. Some cytokines, such as tumor necrosis factor (TNF)-α, appeared early in primitive invertebrates [37, 38] and, therefore, it is likely that the emerging IL-17 gene family may have fulfilled the increased demand for more complex regulation in relatively high multicellular animals. New genes must be integrated with other novel and existing genes to evolve expanded or modified biochemical pathways and/or regulatory networks [39]. Accordingly, the IL-17 family functions via its receptor IL-17R, a specific cell surface receptor, thus forming a distinct ligand-receptor signaling system to induce downstream signaling. In mollusks, IL-17 family genes participate in the immune response to stimulation [19, 23]. Therefore, IL-17 may also play a vital role in invertebrate inflammatory reactions. Inexplicably, other IL members have only arisen in lower vertebrates and not invertebrates, whereas some ILRs are found only in invertebrates [14]. However, why the IL-17 gene and not another IL member was selected during early evolution remains unclear.

So far, five members of the IL-17R family (IL-17RA–IL-17RE) have been identified, and are thought to consist of homodimers or heterodimers. Among them, the heterodimer of IL-17RA and IL-17RC is a receptor for homodimers and heterodimers of IL-17A and IL-17F, whereas the heterodimer consisting of IL-17RA and IL-17RB serves as a receptor for IL-17E. IL-17B binds to IL-17RB, and IL-17C was recently reported to bind to IL-17RE and to activate NF-κB. The receptor for IL-17RD has yet to be identified [10, 40]. Specifically, a mechanism of complex formation has been presented, such that two fibronectin-type domains of IL-17RA engage IL-17F in a groove within the IL-17F homodimer interface [41]. The IL-17R family mediates a signal pathway that serves as a bridge between innate and adaptive immune responses [40]. However, these receptors are rarely isolated from invertebrates. The signal transduction pathway mediated by IL-17 and IL-17R remains poorly defined, particularly in invertebrates, and there is still much to learn about the structures and functions of IL-17 and IL-17R and their characteristics and nature during evolution.

In conclusion, this study provided a global survey to investigate the distribution of the IL-17 family among invertebrates, revealed the features of their motifs and signal peptides. Meanwhile, phylogenetic trees and their exon-intron structures were analyzed, and their origin and evolutionary history in animal phyla were explored. The results of this study suggest that, during evolution, invertebrate IL-17 proteins have undergone complex differentiation, and that their members may have developed novel functions. The findings provide direction for future studies of the functions of the IL-17 family.

## Supporting Information

S1 Dataset Protein sequences of IL-17. (DOCX)

S1 Fig The sequence logos of the three conserved motifs in IL-17 proteins (A) and their IL-17 domains (B). The motifs were analyzed by MEME 4.9.1. The full-length IL-17 proteins and IL-17 domains have similar sequence logos for the motifs, but the motifs have different names.
(TIF)

S2 Fig Phylogenetic tree of vertebrate IL-17 proteins constructed using the neighbor-joining method. The extra IL-17 protein sequences (*Rattus norvegicus* IL-25 (NP_001178936.1), *Alligator sinensis* IL-17D-like isoform X2 (XP_006022303.1)) have not been listed in S1 Dataset.
(TIF)

## References

[1] Gu C, Wu L, Li X. IL-17 family: cytokines, receptors and signaling. Cytokine. 2013 64:477–85. 10.1016/j.cyto.2013.07.022 24011563 PMC3867811

[2] Song X, Qian Y. IL-17 family cytokines mediated signaling in the pathogenesis of inflammatory diseases. Cell Signal. 2013 25:2335–47. 10.1016/j.cellsig.2013.07.021 23917206

[3] Manni ML, Robinson KR, Alcorn JF. A tale of two cytokines: IL-17 and IL-22 in asthma and infection. Expert review of respiratory medicine. 2014 8:25–42. 10.1586/17476348.2014.854167 24325586 PMC4123209

[4] Cua DJ, Tato CM. Innate IL-17-producing cells: the sentinels of the immune system. Nat Rev Immunol. 2010 10:479–89. 10.1038/nri2800 20559326

[5] Rouvier E, Luciani MF, Mattei MG, Denizot F, Golstein P. Ctla-8, Cloned from an Activated T-Cell, Bearing Au-Rich Messenger-Rna Instability Sequences, and Homologous to a Herpesvirus Saimiri Gene. J Immunol. 1993 150:5445–56. 8390535

[6] Yoo ZB, Painter SL, Fanslow WC, Ulrich D, Macduff BM, Spriggs MK, et al Human Il-17—a Novel Cytokine Derived from T-Cells. J Immunol. 1995 155:5483–6. 7499828

[7] Kolls JK, Linden A. Interleukin-17 family members and inflammation. Immunity. 2004 21:467–76. 15485625 10.1016/j.immuni.2004.08.018

[8] Korenaga H, Kono T, Sakai M. Isolation of seven IL-17 family genes from the Japanese pufferfish Takifugu rubripes. Fish Shellfish Immunol. 2010 28:809–18. 10.1016/j.fsi.2010.01.016 20144719

[9] Zhang X, Angkasekwinai P, Dong C, Tang H. Structure and function of interleukin-17 family cytokines. Protein & cell. 2011 2:26–40. 21337007 10.1007/s13238-011-1006-5 PMC4875287

[10] Iwakura Y, Ishigame H, Saijo S, Nakae S. Functional Specialization of Interleukin-17 Family Members. Immunity. 2011 34:149–62. 10.1016/j.immuni.2011.02.012 21349428

[11] Gaffen SL. Recent advances in the IL-17 cytokine family. Curr Opin Immunol. 2011 23:613–9. 10.1016/j.coi.2011.07.006 21852080 PMC3190066

[12] Lee Y. The role of interleukin-17 in bone metabolism and inflammatory skeletal diseases. BMB reports. 2013 46:479–83. 24148767 10.5483/BMBRep.2013.46.10.141 PMC4133834

[13] Kono T, Korenaga H, Sakai M. Genomics of fish IL-17 ligand and receptors: A review. Fish Shellfish Immun. 2011 31:635–43. 10.1016/j.fsi.2010.11.028 21134467

[14] Huang S, Yuan S, Guo L, Yu Y, Li J, Wu T, et al Genomic analysis of the immune gene repertoire of amphioxus reveals extraordinary innate complexity and diversity. Genome Res. 2008 18:1112–26. 10.1101/gr.069674.107 18562681 PMC2493400

[15] Takeuchi T, Kawashima T, Koyanagi R, Gyoja F, Tanaka M, Ikuta T, et al Draft Genome of the Pearl Oyster Pinctada fucata: A Platform for Understanding Bivalve Biology. DNA Research. 2012 19:117–30. 10.1093/dnares/dss005 22315334 PMC3325083

[16] Simakov O, Marletaz F, Cho SJ, Edsinger-Gonzales E, Havlak P, Hellsten U, et al Insights into bilaterian evolution from three spiralian genomes. Nature. 2013 493:526–31. 10.1038/nature11696 23254933 PMC4085046

[17] Zhang G, Fang X, Guo X, Li L, Luo R, Xu F, et al The oyster genome reveals stress adaptation and complexity of shell formation. Nature. 2012 490:49–54. 10.1038/nature11413 22992520

[18] Hibino T, Loza-Coll M, Messier C, Majeske AJ, Cohen AH, Terwilliger DP, et al The immune gene repertoire encoded in the purple sea urchin genome. Developmental biology. 2006 300:349–65. 17027739 10.1016/j.ydbio.2006.08.065

[19] Roberts S, Gueguen Y, de Lorgeril J, Goetz F. Rapid accumulation of an interleukin 17 homolog transcript in Crassostrea gigas hemocytes following bacterial exposure. Developmental and comparative immunology. 2008 32:1099–104. 10.1016/j.dci.2008.02.006 18395796

[20] Wu SZ, Huang XD, Li Q, He MX. Interleukin-17 in pearl oyster (Pinctada fucata): molecular cloning and functional characterization. Fish Shellfish Immunol. 2013 34:1050–6. 10.1016/j.fsi.2013.01.005 23357025

[21] Petersen TN, Brunak S, von Heijne G, Nielsen H. SignalP 4.0: discriminating signal peptides from transmembrane regions. Nature methods. 2011 8:785–6. 10.1038/nmeth.1701 21959131

[22] Tamura K, Stecher G, Peterson D, Filipski A, Kumar S. MEGA6: Molecular Evolutionary Genetics Analysis version 6.0. Mol Biol Evol. 2013 30:2725–9. 10.1093/molbev/mst197 24132122 PMC3840312

[23] Wu SZ, Huang XD, Li Q, He MX. Interleukin-17 in pearl oyster (Pinctada fucata): Molecular cloning and functional characterization. Fish Shellfish Immunol. 2013. 10.1016/j.fsi.2013.01.005 23357025

[24] Flajnik MF, Kasahara M. Origin and evolution of the adaptive immune system: genetic events and selective pressures. Nature reviews Genetics. 2010 11:47–59. 10.1038/nrg2703 19997068 PMC3805090

[25] Nehyba J, Hrdlickova R, Bose HR. Dynamic Evolution of Immune System Regulators: The History of the Interferon Regulatory Factor Family. Mol Biol Evol. 2009 26:2539–50. 10.1093/molbev/msp167 19638535 PMC2767096

[26] Yao P, Fox PL. Aminoacyl-tRNA synthetases in medicine and disease. Embo Mol Med. 2013 5:332–43. 10.1002/emmm.201100626 23427196 PMC3598075

[27] Hartupee J, Liu CN, Novotny M, Li XX, Hamilton T. IL-17 enhances chemokine gene expression through mRNA stabilization. J Immunol. 2007 179:4135–41. 17785852 10.4049/jimmunol.179.6.4135

[28] Chiricozzi A, Nograles KE, Johnson-Huang LM, Fuentes-Duculan J, Cardinale I, Bonifacio KM, et al IL-17 induces an expanded range of downstream genes in reconstituted human epidermis model. PloS one. 2014 9:e90284 10.1371/journal.pone.0090284 24587313 PMC3938679

[29] McDonald NQ, Hendrickson WA. A structural superfamily of growth factors containing a cystine knot motif. Cell. 1993 73:421–4. 8490958 10.1016/0092-8674(93)90127-c

[30] Hymowitz SG, Filvaroff EH, Yin JP, Lee J, Cai L, Risser P, et al IL-17s adopt a cystine knot fold: structure and activity of a novel cytokine, IL-17F, and implications for receptor binding. Embo J. 2001 20:5332–41. 11574464 10.1093/emboj/20.19.5332 PMC125646

[31] Walter P. Signal sequence recognition and protein targeting to the endoplasmic reticulum membrane. Harvey lectures. 1995 91:115–31. 9127989

[32] Le Hir H, Nott A, Moore MJ. How introns influence and enhance eukaryotic gene expression. Trends Biochem Sci. 2003 28:215–20. 12713906 10.1016/S0968-0004(03)00052-5

[33] Swinburne IA, Miguez DG, Landgraf D, Silver PA. Intron length increases oscillatory periods of gene expression in animal cells. Gene Dev. 2008 22:2342–6. 10.1101/gad.1696108 18703678 PMC2532923

[34] Gruss P, Lai CJ, Dhar R, Khoury G. Splicing as a requirement for biogenesis of functional 16S mRNA of simian virus 40. Proc Natl Acad Sci U S A. 1979 76:4317–21. 228296 10.1073/pnas.76.9.4317 PMC411565

[35] Palmiter RD, Sandgren EP, Avarbock MR, Allen DD, Brinster RL. Heterologous introns can enhance expression of transgenes in mice. Proc Natl Acad Sci U S A. 1991 88:478–82. 1988947 10.1073/pnas.88.2.478 PMC50834

[36] Jeffares DC, Mourier T, Penny D. The biology of intron gain and loss. Trends Genet. 2006 22:16–22. 16290250 10.1016/j.tig.2005.10.006

[37] Hayward DC, Samuel G, Pontynen PC, Catmull J, Saint R, Miller DJ, et al Localized expression of a dpp/BMP2/4 ortholog in a coral embryo. Proc Natl Acad Sci U S A. 2002 99:8106–11. 12048233 10.1073/pnas.112021499 PMC123028

[38] Technau U, Rudd S, Maxwell P, Gordon PM, Saina M, Grasso LC, et al Maintenance of ancestral complexity and non-metazoan genes in two basal cnidarians. Trends Genet. 2005 21:633–9. 16226338 10.1016/j.tig.2005.09.007

[39] Putnam NH, Srivastava M, Hellsten U, Dirks B, Chapman J, Salamov A, et al Sea anemone genome reveals ancestral eumetazoan gene repertoire and genomic organization. Science. 2007 317:86–94. 17615350 10.1126/science.1139158

[40] Gaffen SL. Structure and signalling in the IL-17 receptor family. Nat Rev Immunol. 2009 9:556–67. 10.1038/nri2586 19575028 PMC2821718

[41] Ely LK, Fischer S, Garcia KC. Structural basis of receptor sharing by interleukin 17 cytokines. Nature immunology. 2009 10:1245–51. 10.1038/ni.1813 19838198 PMC2783927

