# Supplementary material for: Comparative and Evolutionary Analysis of the Interleukin 17 Gene Family in Invertebrates
Source: PLoS One. 2015 Jul 28;10(7):e0132802. doi: 10.1371/journal.pone.0132802 (PMC4517768; doi:10.1371/journal.pone.0132802)
Supplement: S1 Dataset — (DOCX) [file pone.0132802.s001.docx]

1) IL17 protein sequences that contain the complete IL-17 domain. The IL-17 domains are marked with the red font.

>C.briggsae_XP_002637129.1 Hypothetical protein CBG09631

KEELSAWMQISMNGQFDDMALEEWSTNGKEPEICGKSPRAEGSSTIMERALCPWESRVNFQESREPKLIAESVCLCRKSRGSTGAFCMPIIRKVPILRRISCDRSTGLWNYVRSTELITVGCHSVLPRTQRNVRLANLSSDRVVV

>C.elegans_NP_505700.2 Protein F25D1.3

MICFRTAFILILGVVIGTTAKRHNVRRHVNGKWTDKHCEEPPSLKEDLATWMQISMSGQFDDTALEEWSTNGKEPEICEKSPKADGVTTIMERALCPWDSRVNYQESREPKLIAESVCLCRKSRGSTGAFCMPIVRKVPILRRVSCDRSTGLWNYVRSTELITVGCHSVLPRTQRAARLAHLSSSRIIV

>C.elegans_NP_510131.2 Protein T22H6.1

MPKSPHRTNLQSFYPIMIILTHLALDSNCWRIRREVDGEEEDIMPSEECYHKYNEENHFKVFSNYLNRKNSSHYSPIAPSYQQALLRLQVKGLKHGEQITKSSGKCNSKKLDTISAETPLRDRALCKFEYVLNYNPKRLPAALTEVKCSCPRPNSKLVGKRIFECEHLRYQVRVLMWDDSCNTFREHVETIALACIPVIQANANADGDDDFIYTIKAEIPI

>C.teleta_199819

MQILFQLGPSDAFQSTVGNIFWVREGLESHDSCTAIQQTKAGRWNMTSYPLNSLKRVREFYSFSSERVCVLFVMVALVTSCMEPNLTSIWQTTLYQNGINNAQNQQGDYAPVTECRDHCDWSSELGCRVCETVEGQRDCSAPSTSGSLNRKSFCPWYYVSTYDERRYPRDLFEARCSCQKCGINNIFSCEHVHHVTPLLYKTGSADNDGYCIYVPRLTRIAVGCACALSKSQN

>C.teleta_198235

MGIQQYLIALLFICCAVTLVNAASTRKGSSGESSNNYHRKFVVHKILGMVHSKSQQANQGMKTTEMSENKEEFLVEQVSGSDDQWPFDGVVPPMSVDMMKMQEEYASPDEDISVAQTLLDLTQMNRKNSVIRCNEVFSIKDEKTRNALRHLCNERFGSESTVDPKSSSLSETSSTTDALDLNSSGASATSTSTESSATSALSSTADSTVSHTPSSIILSSITATSDSQSQSSNAGSTTTAQGTTTETLDATAPDATTSGIKTTEDASETPRSLTRRKRSTSSILLCPWTYEINVVPHRFPEAIYKAKCKVSQCQRCTEKGGYFEEFSYRMQVLSPDPSMPTEDKYKYIHSSETVPVACVCRLPHNF

>C.teleta_206957

MALCECILLFWTLLTVSVNACNDTPEQDLKFQWMRSLENTCQHSSFLMAKPFLELSQNRSDIYTGRQFQCPEFTGCTPPGHDGSCTSCVPAVQVETMDGLPSLDATDDLRLRSMCPWTYIETKNAKMRYPPLLVEASCLCRPSGACPCGPRGSVDSSCEAVDVPVPILVRTTEVDFEGRCVYKPQMYRLKVGCTCAMTPVAH

>C.teleta_216301

MKYSFLLSAFVAVFNLGVVMISARSMAQCKERSDRQLLTLWQHHASVHNASLLQVPEYSHHARTSRHRAAAFPLTNQPTCSGSHQIAELSERSVCSWHHVITSSPDRYPKDMVEARRAAECGRHCLGRQGRCVELRYPVAIIKKTGRCDHEGFYAYEARWHPLVVGYTCRARSTMATYAA

>C.teleta_205055

MVILTAHQSSPAPLQCRERPRDHLHQMWSTYSTSLGIHESSFAVKEMPGNFSRHPYPQSSPLSSGLNSCNPGNHVTQRVHQRSHCAWSYALSYDPDRYPNLLVEAVRPSDCVYCLGDGICEAMTYPVIILRKTGQCDAAGFYEYVGGWLRLTVGFTCAVRPEVIAF

>C.teleta_209749

MKAFVEAEFAAKIKDHSGLEADYRGHFICMPMDVYLSELNLDAIAEQSWNSEFTLFIRCRIFYILSLCVRKAINGYHRQQRPVHACGDPIEQLPKPMLAFDFNDSYDTRVLSFCLAKCSHESKRLQVMQGTKFTYPKSNQFLDRYWKCSGALFTTYLYGGFQLLQLTFFEVLRVVTASLLVGQSHSASFRCTEPSQKDLHAQWTAFASSETAFAHPSVYLLPEYRHHITPSTFDSQEFALDAPSGDVCAVSDDIRSSVARRSLCAWYHVISSSDDRYPRDIVEAHRPDECGHRCGLSGGVCAELTFPVAVLRRTARCDEHGLYVYEGAWHDMVVGFTCTAAPEATSFA

>L.gigantea_152638

MTMLVILCVLLGLVDSKSLEKVLLNQKSRNADDCQIPDNITELIESLTEIKRNKSFLIPVQESIYEVCDRRKSEIPFVTPTNCPENVTSRRGGKLNQRSVCPWDMVQEYLPGYFPSVINVARCRLCERCSGVNSTTSCQPIVYRIPVLKLTGCVSGFYTYEKKYKKRAVGCTCARRKTMRARRRNRV

>L.gigantea_169526

MSFKVALTLLLIIMPYFLKCLSEGRTIANSHRRTQCKMPKNLCQLRKEVIRPAALRDAFSIDLINGKNITDVPPEYIDTHHYVSGGSKICQKSSDNELCPVYYVLNVDENRIPNSFLEAVCSCAWPQMDILKDTRVECQTKNYYTKVLRRTGCDEEHNTFIYEAVWEPVRIGCVALSGPERYISLQPVSPI

>L.gigantea_164174

MFLKYLALIALIAVSLLITNSTSQFQTGRCREPDPNELLRILKSPDPETLPPSFMMLPHFRKLSTTHLPSNRSVNVLFRGETSCKKMTANICPSYHVVQYDPERVPSTIIQAECACKHCKSSRSFKRYGNGMKFNCEKIISYSRVLRKTECKLNIDTNDLVMQYKKVWEPVSVACTCVLK

>L.gigantea_228210

MMVLMKVFICALLVNMVYSFTLIKNIEHILERERRSDECAVPNNIDELFTNLNSHVNRVEFLIAVNATHQPNAQGFIPPAFGDQTDTECPKEYTGKDTSNSEIWQRSTCPWFYSERNYGAEYYPSKVFNAACKCVDCLYSKNNFETGCSRIYKNINILKKTGCSNGFYTYEQRSISVSVGCTCAKLQANIG

>L.gigantea_172928

MTANNFLTILIILLWNWSDSSEGHVCLQSTNLIESWRNWDIEITTERLRESGLFNTCPSMLALGPFSPDQDRSSCPWYYVNNRDRHRLPSVIQEARCNCTRCFGTNEDSYCREQYVNVPVLFCFPLDDSQCSVRFDRISVGCSCYFPRRLENIR

>L.gigantea_159302

MAYKLFFVAMVSLLSSGGRSMPIDNKTCVDPDMSKIKLDVDELHDSFYQLPSMNDHIVNTTSASPYIATHRSYMHGPKQCPTIEDIVNRYDGHICPVYYILNHDENRIPKSFVEAECSCGKPPIQIEGGVLLLECAPLYKYTKVQRRIGCDNDGFYIYTPMWEKVKIGCFTQLPLGFIGDGVGTPY

>C.gigas_ABO93467.1_interleukin-17

MGNFFLFAMTLVVCSVIVLLTGVADSAVICSEPTNLAEQYSQYMANATVSNMLEYLESSTAGGVASDEEEQLIYGNRICPSSIKAITTDPKSSVFERTTCPYFLVASHLSTRYPKIITEARCKCSGCVPLEENGHSDLTRCEPVYRPVRVLSRTGNCVNGVYQYAAAVHMKQEGCTCVRKTEALSGSGSGSNGSDDPIPM

>C.gigas_EKC33705.1 hypothetical protein CGI_10020734

MNLKLFILMTLNYLCPMMAMVIRCNLPLTLGYSKNEKDIYQESRYILPNTHGIRPGLGTLYSIDANFINIQYVNHSEKTTCEEMSSSTRNNYHITLAESASCPWYYRKNIDENRVPRELMEAKCACRCSLHRPNCARATCREVYRYVKVLRRSSCTEVVEVIEPISVGCTEFIAEQIIFENMRWISWNNS

>C.gigas_EKC26195.1 hypothetical protein CGI_10027182

MGKLNFLLLLAISDIWIIPVATLCRDPESLNITTLQMDVEKKLTLRNVPNSFVEKRTHTRRFNARCPSKTDVLSGPKFRTSICPTYRVTDVDVNRIPQTIVQRRCKCTECLSVLDSTLGPRAFSRCVPTFQYQMVLRRVGCASGVFEYKPVMEPFVVGCSCKLFFDQ

>C.gigas_EKC33786.1 hypothetical protein CGI_10014828

MARGLSRYKLKDLPESFYLMDKLRNKAIVPPEYSESIYVKVDNESSTCPTTLDNKLHVCPGYKVMEYDRFRFPSMMLQVHCKCNGCLGSPDKACVRLFYYTRVLRVTGCNKKGVYVYDYFWEKVSNGCVCIKNDRQTMSRG

>C.gigas_EKC38792.1 hypothetical protein CGI_10026592

MIASLNTKTDKNCDCQTRSENKTINISGSRNCSLKELSRKKVVDSCPYHYQLNYDRFRIPQSIIQVKCNCDNCIGRKPDGSLQELHDGRCTEVMIPRQVLRLCRKKRRKVYTVRIEEYAVACTCQWPNPNQV

>C.gigas_EKC33462.1 hypothetical proteinGI_10015251

MREIILTIGMLMLLPLPTRGRSIPQHFNPAAEWFFLQAIGTAHQCRADFVPALQCPASPLEHSPFDDNEFLGKRSTCPWVMCRDSNSHRQPRDIYYAKCLCTGCKGQSGEYSCQPIYTVVTVSRVTSRGIHDDDSLRVPLGCTCAKNR

>C.gigas_EKC32654.1 hypothetical protein CGI_10004922

MNAPVWLTLVLALLTRCCLPHPVSRCRGCHIFDPPFRGLDYSDYDDSERLDTQSICPWSTIMVEDNDRVPRQLPSTTCTKSSVVFQLNGTPVEYACELVEVTVSVLKYHPSGYWERSDELVPVACTAVQRPS

>P.fucata_pfu_aug1.0_1712.1_51392.t1

MAQQTQGINYSDYAATPTRFHCNSSAFYGITLRPYRISILVALGFTIYLPSICIPCQEPSTYPQIISNLSHESNLISQRRALQNGIGTIPSEFLNVSILEIHQHRRNTRRISNWQKVVQGSTCPWKFVINFDPNRKPSALTEAQCTRQCCNGDECRTTRCEELKYELPVLRRVGCRHGVFVYQQVYETLAAGCTCVYAVDDAFAMPIN

>P.fucata_pfu_aug1.0_1712.1_51391.t1

MLKFVLYYVCHLPCGARKKAPREGFENFINITYENISNLTTCEDVRTYRITNVMSNHQSIMATCPYFFRRNMDPNRIPYSLTEAKCACQCCGMESRCNRRQSKCREVYRYADVLRRYNDRGHYKRVLEPISIGCSCINPIPRRTLRQRINLEMHMPEK

>P.fucata_pfu_aug1.0_1712.1_51394.t1

EAFEIILLLILLVNLVLTENNQCNLQQKDLIKNSIYDFHKNVDWLPVILNLSSPKGCIAPRNGTKSKKPTYAHFMRRSCNRTKRGNRLRSRTICPWHYVLDYDPYRMPATIYQAKCTCPGKKRNGCKCKTMKLKLPVLRISVFDGEMEPSVERISVACVCEMRGKNQTS

>P.fucata_pfu_aug1.0_204780.1_72074.t1

MSFQTSMLLNTVLMMFALINNHNVFAHSSFAIRRIANNMCLLSDEPMEVFFDPIEPLNENAVCPWTIDYDYDPQRIPRKLPIATCLTDKGIIPSTPACNSRNPVVCDQIELDVPVLISHDNFPGVFEEKLKRVSVGCAGRLYPDK

>P.fucata_pfu_aug1.0_20923.1_18751.t1

YIDAYTSKLEDDTCPTKKQQVACPTYDVIDVDKNRIPEVIPQKRCRCRACLSNEGDVCMQVYTRQTVLRRVECHNNTFIYRPALEPVVVSCEWKRHLASDSNFTYRYIDDVFSINNPKFADYLSTIYPSELEVKETTETNNSASYLEIILSYDTDGHMNTSLYDKRDDFNFSITNFPFLSSNIPSSPAYGVFISQLIRYARASTKYTDFVLRAKRLSDKLLSQGYVCDRLTSSLRKFYGRYGELFIRYDVPLSRMVDDILS

>P.fucata_pfu_aug1.0_24776.1_26199.t1

FIQGRPQNDQAACPESTTPTRTLTNGIIVFEEAQTGDVPTTTDTNTYIEPKNSEKLCPTQLLNKVSAELKDRSLCPWFTVLNRNQFRVPRIIKEARCRCSKCVVPSSDSFAGDCQCEQIFENIKVLKRTAKCSNGQFVYRLQVERIPVGCACAIRRQAMSSESPA

>P.fucata_pfu_aug1.0_27731.1_19195.t1

VAGPALLLCLIYVFEIRGAPIDCAEPENLNARFEELNNGINITQFYFPPSLSENLVNESDAVLKYIKHKEQQFGIYGGTLCPASLPTDGDYPIEIRSLCPWYYQSVHDPAYFPATIPEAVPRCQTCVGHNESFVCERFYQRISVLKKDGCVEGLYRYVELQKDIPVGAVCARRREVESSSSSQDISMKK

>P.fucata_pfu_aug1.0_27889.1_19207.t1

MQMRRIIIVMAYVICILDIIEYVNCSIRRKELVLMEEANYTMNVFRKQSCRASYTSNATFSMITTHNWFGLRHYSRRAKCSWGYRNNTDMSRIPETLVEAFCIQPYCGRSTHSSRCRADCLCQEVDRFVKVVRKDAPFEEDWQALTVGCTCMCDTLTNMHVMPIY

>P.fucata_pfu_aug1.0_32457.1_48078.t1

MVNVNPNLRRCIESWNESSDFRSQLMQLRNSLHPMDFNARSFGPNLVANSRCNRRAIATPVFQNERSLCPWEIAEDYDENRYPQLLHYARCKCRYCQGGIRFECMPLSYRIPIIERQCNNGVFEYVKTFADLPVGCTCSRPYSIEM

>P.fucata_pfu_aug1.0_8564.1_24423.t1

MCPWRYDIDHDVTRFPASIPRAVRKCLTCVGLNSSQHVCELISQTITVFRQDGTEANGSCKYRQISESIPIAFVCAHRREVYSGPNTLEDPGMISM

>P.fucata_pfu_aug1.0_8564.1_24422.t1

MTYKEILSGFFVYCVTYAFCHRLYCLEPPNLRQAFMEINYNSGSGSGYFPPSLIPEYTRDPIPLAKRDISYYRDFPVPKRDVSEYSCPRTSSASDLLRDRSLCPWRYIPDHDSNRFPATILEAVPICPYCTGIGNVCERLFRSLKVLIRRTDSCFNGTYQYYETTQRFAIATLCARRRETAHTRNGFLASRRRTRIYSGPNQAVSM

>P.fucata_JX971444.1_IL-17

MYKIILLVLLGAFGSFVQSSPLPPCQEPENLDDLFKNLTYQGNMDFILPPFMNEETNVIPPPEEVQYLTGLRTCPGGTKDLEVGMDTPLSSRSTCPFYFVTTHDSRRYPASITEARCSCTSCLDFDGPSPRNKCEPMYRSIKVIVKHECVNNIWKYKVATYLKQESCTCALPREVKNGQQESSSGSETGDPEPM

>D.pulex_125692

MDTPFLQHNKQKEHIAKQQQSTQPIMVTISIVLYLVILLAALSGLASQSSLKANLKQHSNHHTGKSVKKASSLLNETKRLQRLLDEQFSKFLEQLPFDLSFLQLEENQVKHARYIAKEQDLSAAHSNEDCNKPYELEATVEEFRTCPSQLVAVKRQDRFPNVRLFAKCLCRKCLGNTITSYPYSSSTCLPVKVLMPVLIRSHSSGQQSDAEWKFFLEPVSVSCVCGTKQRPDN

>S.purpuratus_SPU_019350.1

VYNIAILMIIVVVHAVTSSPVKSSSQYCLPMNQADYESRERNGHLFYPKQDAFAVQSFNVSDADMGRIETSSCPYDGFSSVQGCPAGAKPSASEPVNSNQDGMCPWTYVECFDSDRIPMSISMAQCQCSACLDPYSHEADPNLRCQPIFHNMKVLRKTQCVDGLYRYEEETVKVPVYNIAILMIIVVVHAVTSSPVKSSSQYCLPMNQADFESRERNGHLFYPNQDAFAVQSFNVSDADMGRIETSSCPYDGLSSVQGCPAGVKPSASEPVNSNQDGMCPWTYVECFDPDRIPMSISMAQCQCSACLDPYSHEADPNLRCQPIFHNMKVLRKTQCVDGLYRYEEETVKVPVYNIAILMILVVVHAVTSSPVKSSSQYCLPMNQADYESRERNGHLFYPSQDAFAVRSFNVSDADMGKIETSSCPYDGFSSVQGCPAGAKPSASEPVNSNQDGMCPWTYVECFDPDRIPMSISMAQCQCSACLDPYSHEADPNLRCQPIFHNMKVLRKTQCVDGLYRYEEETVKVPVACGCMRQRVSY

>S.purpuratus_SPU_022838.1

MIVAASCIQIAFSSPLQWPSAGAGLSGVPDGQGQTCLADDLEERARRQEVAQSLYPNQRAYAVQSFDDATPDANSDDVPSANCPMDGLGGQDTHPKGMVPSQGRDVSTESTCPFTYVTCYDSDRIPARLTVAQCECSACLDPYTNKEDPELVCQPVYYNIKVLRRKSCKNGMFHYEIVSSGLAMSFVLTLVSSNPYGRFRKDHPDLWAISDTITNERRRAFDPTAEADALHQSFPVTANSLVGANNNCPTTELSSEECPDGANVTHPDHHNENSVCPWTYIHCSDPGRIPEVIAVAQCRCSTCLDPYTHRPDQNLVCQSIMYKMKVLRRTPHASGQYRYHVATEDVPVACACLRKRTSVGRSGADNSSSVVTEVVETLD

>S.purpuratus_SPU_030196.1

MAQDLFITFAIAAVSCSLMTSRVISSPTIDRANHGNVTCREPNTTELTSMLALAVARNPTLAENARALFPGLLDSSAEAILRSYDYNFTTHLHPMSKCPRHFLNSMDRAQEVRSLCPWRYVLHSDNNRYPRDIIFVQCECQECVDPELGVFSSNRDLCRPVIHNHHVLRRTGECINGVQRYEEQFEPVPMACVCERFASRMSIN

>S.purpuratus_SPU_030199.1

HYRPLHVECAFIGAECATSNTHILARIHKGGFRTSYDAFLRVFYKPGLLRTKGGLANQKENTKTVVFFLALIAASSCNPVPLTCIPGQSLARTDLYPNKAAFAVQSFSLSPLDIGRSQSSTCPNTTFTGISSNATCPSGTAPGSTLVVNENGLCPWTYVECFDADRIPMGLQVAQCQCSGCLDPYTHTPNPNLQCTPVKRNIKVLKKTQCAGGMYKYEEQNLAVPVACACMRQRVA

>S.purpuratus_SPU_030204.1

MGYKLKKYSKNTNPNYLYESADIYVQRSLIMKLELMMTVLCLTAMSISNPVSSQHCPVSRTEYLPSYEDSDLLLRTDSDAVGVHPRNVLHYQSDVEQPASELQCRDPLSQPGALRSPQNAGLCPWEYIADHNATRIPQTIYKARCLSCTGECINPMTDSSSTIHRLFSRCSEVYHNIKVLVRSANSCIGQTGLLQYEEHVLSIPVACVCTRFGFL

>S.purpuratus_SPU_030198.1

MKMYQPTHVVLLILSAMTSQCLAFSVGRHHSDVEVVSVIQSSLPSYNTDVKSNTDSLGQAFLVNTKEIDASGQGISFDGDQEGNGISSESDLYEEIHEAVYLTVKPNSKDNPEIDTNSGNTLSITSSRIELNTAFHLDATSVESATAVPFTSGSGENELVEVASRIHFTTQASDGATGSMRIKRNADAPNLDHADTPTEEADATATEACRDPSEGELYEMLHGQTIAGSGTEIQHSYLNNTCPASLLERNQKPLESRALCPFVMETDTDVERYPQDILSARCACPDCINPYNNGFIRNPGVDCMPVVREMETLRRGQCVDGVYRYEKQTTKVPVACVCARRRAV

>C.intestinalis_XP_004227512.1_interleukin-17D-like

MESITKRTTLFLVIAICVKYSLHAPVNHVTAQVTPYFHDNIVAQINRTLPLQYFHHIKQEKPPRGWARRCEKDTHECPENNISLLRELGTHPSVSKRSTTPWKYVLNQDTNRYPVNIYEACCLCSNCLTVMDGRHIPDVKAAEIKVPIKAAFWNGTGPVVRYIHIAIGCTCVRRRSSMG

>C.intestinalis_NP_001123348.1_interleukin-17-3

MINNTEGSPILDLLQYEEKDGEYELVKKSIPTLDKIKELCGDDEICIRYNEVYAQVLNDTEYASQFTVIDVTNDKSQNGGKKQRTKRSLQCPTFNPASTSVEQRSLSPWTYVIHQDRNRRPSTILQAKCLCYGCYDMDSPTLAENINLVSTPIRYSVRLPKRINGVWVRRPYNIQRGCQCQVPLYM

>C.intestinalis_NP_001123346.1_interleukin-17-2 precursor

MFKLFLLVLFVCVCYGNEVRYRRNEKRCRIPLNSQNRKQCRSIMKQAYVQYSYKLSRFDQSALKNSKMGTSTAKCPTMTESYVINENSPTARSTSPYVTELVYDNKRIPRYLPQSKCLCSGCIASSNGRETLVGKSVPLVAQIKVMRRNKTNSYSVVTEDVTIGCTCVLNV

>C.intestinalis_NP_001123347.1_interleukin-17-1 precursor

MTTTMQILIVLCAVAAINATPVLIKRFIEDLKCSSSVANKEICDLLKKQSAGNVTENQHGIRHKRSQSTCPVLSDEYLLTHTRQEERSLSPYVIEDDIDRLRIPMVLPRARCLCDGCIDMSSRRENFSFASVPLKQSFTVRKRNTENGPLETVTQTITVGCTCVVPRRISQ

>C.intestinalis_203738

MNNLKVSLICACALILRSMANGTFVASSLKYDFVNGNPVLQKNSIPTDKDIVQACGGDETCEEYEKTYALVLKDNAFAKSLEMVDVTPGEHSGDGEVREKRSPSCPAYDDSMTNISRRSLSPWTYIRNVDRTRIPVVMLDAVCLCGGCYDISSPSLAEDINLYSTRVPGTIPVRRIRPGKPSVPDYVNLHTGCRCVVPL

>B.floridae_91950

MSPRNDNVVKLDINRTARGRFGAQTKEKAKTAHPVRHSYSSDDSTEMTVTTGDTAVLDQVEGFIDTWEGAERNVSRDVSRAIRRGHRPPRRGRKRNGAHPLGFHLETEQSNMNENEFTTDKTTLSKKTKEDSERDKERMKRRKLKKRNRRCRRRRKKIGSLKRTARQGPKSKRRKSASFENDPKQKQRKDTNNTNPYSIGHFKEQEHRDDGKISNFRNVNVTSQHGLGTNSSMVEPLQTINSTRRHSSRLTARKRRRHYFRKRKKCRRNDRKKGQMRHEKPHITETFREDKVEVTPVTSSHNKSFSRKHGVFGNVSNNPKEDVLQKSPRETCPENRTTDSPNLPLNERSVCPWSYYIDHDPNRVPHDIARAKCRCTACLDPVTKKQNYNYACVPVTIQKLVYRRKKKKSGGYRYREEWQGVTVGCTCVQPRYSP

>B.floridae_117645

MASFIVALFSFLALGGVLVSGVAIPTTDTYPTDATDTGAQARSGCSEPSAAELSQLLNDCSSANNPETKELSSGPNPCESGTCLQPAENDLSQRAYCPWQVIVDSNPNRFPTDIAYARCQSTFPSQDGEYNWTMACDSVTYTKPVLVREECSGADNTYRYKCVHLTVPNACVAVEPL

>B.floridae_230778

LNERATAPWDYVIDHDPNRFPSSIPQARCLCYGCIDVNRGVEDTKLISVPLTYTTKVLYRRGCDSNGRVKYRAREVKVKVGCTCAVPRQGIVG

>B.floridae_127768

MKIYAVITVAVLLLALVSAIDGQKRRSKDRRREGGDKERRRQSKNKGNELETDQCEDISDKQKQRKLRGARKAYERTAPIVLAEQLKKDAGRRRRNAEMACPESEAPHDGDISQRAISPWAWELDVDVNRYPTEIAKAKCLCTAGCLVNGKLDFNYASEPIVTQMKVLRRTKCDRKGKWRYEVQWEDVPVGCTCASPKS

>B.floridae_92872

MVRGVVILLVVASFVDHLRPTQGNPLLPRVDKQGDTTGLLSALGTIRRRHGLRKQGDRPVLVSALGPSPVHPPRHHDRYLSQPPVPVDNPGIERSMCKWRYEDNVDPNRFPSTLKVAVKEYTGSRCRDPATGAPRADLACLPIDYELNVLRKNSEGEWQESYEFVTIGFTCAGSEPR

>B.floridae_132638

MRMRLSVVIVMVLVIMLATEQEARGARKRGKGKTAKKTRPCRKERNKQWCNKDNVEEMLKNHTAVHKENIKKALEETEQQTCPAGARNLRDDDSRDPNERSLSPWTYVKNESANRIPGTYVEAKCLCEGCLIYGKDGVSVENTKDYESLPIKTSLPILTRYDCKGKKCRERLEIAQVTVVRQVQGVVRLGMVGRNEPAHIHRWPTWTWLTRATYSSDHKDTLSKSELKRRLKAQQKQKEKEEKAKAAAEKAAEVVAKKAAGPAQDAIDEESLDPNQYFKIRSQAILDLKKGDEPPYPHKFHVDLSLKEFIDKYHHLNAGDHLTDVTLSVAGRVHAKRESGQKLIFYDLRAEAMKIQVMANARFYTSEEEFFKINGRIRRGDIIGVIGNPGKTKKGELSIIPTSIQLLSPCLHMLPHLHFGITNKETRYRQRYLDLIINEHVRDKFIIRARIINYVRRFLDELGFLEVNFN

>B.floridae_94821

MEHPMIVALVIAVMTSPLMTSARPTVHRIDVDGSLSSDLDQQFRTEDIPLSGTDPTSAKALQQASEVDETKREPTVSTTENLREKFLQLNNFDRLEGEASSDGLLARLAHDQVQHPVYGRGDTAVHSRRARDVTDSGSGSDSDDLPAHYRRHLSQPPIPFDDGANIQGRSVCPWRYDDDFKANRFPHTLRVAVKTHTGSRCIDPATGAPRRDLRCLPVEYKLNVLRKDSEEVWQISADPEFVTVGYTCARSRTA

>B.floridae_66165

MAKVILLLVASVTVLSGIIEGKTRCRSTMKRSDPGPGGYRVFNRKQLAIITPLLNLREPKRNLNKSLNKRTDAPFRMVTDCNRDRIPRDILVARCREEACDDDASWVKGNTYASKLVVWKVRVNGKLQYHAQREKVPVACTCMRPLVAGPG

>D.rerio_XP_002666482.1_ hypotheticalprotein LOC100329556

MMLVCLLVLLVTGLSSAERYPCSTCNCTAAEFLSVSNHLIYPILNYDVSTTSLSPWTYKCTKDMDRIPMVLCNAVCDQPCWKFSKAAKPAGIKVRISTFRRHPCDNGRYRLSRTQYKLIVGCTCVK

>D.rerio_NP_001018634.1_interleukin-17a/f2 precursor

MFLNFFSAKYLVLLGCALARLTIAQQEQNRLCDTALTISNDFNGSQSEDGKGNGSIHNRSLSAWNWIPKFSPHRIPQVIFEAQCSSEYCILPTGVDKRLNSVPIYQDILVLKQEMERKKCFRAMFEKVIVGCTCVRAKTS

>D.rerio_NP_001018625.1_interleukin-17D precursor

MRAPVVSTLVLVWLWCCGSAPGERGQKVSRRAPRTRSCLDLPEEILEQMFGRLSVGVLSAFHHTLQLATPERQNLSCGSASRLARDRPRTPVNLLSLSPWAYRISHDPARYPRYLPEAYCLCKGCLSGLNGEESDRFRSTPVYMPTVILRRTVGPCVGGRHSYTESYVSIAVGCTCVPLLEKDSKIQKSKPSVRRVASKNTVSSTFRKNELN

>D.rerio_NP_001018623.1_interleukin-17a/f1 precursor

MSSALNLRFLMVACMMGLVLISFGAEGASVRSDQKNKNSHPEADHSYRLVLDAEFKASTNPIHPINNDSISPWTYMFTHNESLYPTSIAEAKCSLTGCLIDGVEVQDYESKPIYTQIMVLRRIRGEKPNYSFKLEYKTIAVGCTCVRPYVEQL

>D.rerio_NP_001018626.1_interleukin-17a/f3 precursor

MRLSRVFRAVLLLFLLMLLLDAALSENRTKRKRCSGVKKCTSAGCKSRCQRKTAWVILNSAWDNIMSDTPSPDRSLSPWTYTTSVDESRIPSTISEAKCEKRGCLTKDGEEDLGLESQPIYYQINILRRVKKKNSTFYALKLETKKVSVGCTCVLPIVLPQN

>O.latipes_NP_001191715.1_IL-17A/F-3

MLLVLRALLLLGLLSLEHAKKSQTFPARLKQGPKYRTLKVSLDPSVMPQFYSVSTSNLANSSLSPWTYRENYNSSRLPKSISEAECQTSGCIRDGVEDDALEAKPIQYQILVLYRVQKQQSVGKKKKKKSRKYDFMLGTQVITVGCTCVRPSVITQQ

>O.latipes_NP_001191713.1_IL-17A/F-2

MELPTHSICILMVICCSLRFSSCSDEGVLHPPDCNVTLQFSSEIFSLSRGNGNIHQRSMSPWRWRSTTVRHRIPSTLWEAECDSIFCSNPTSGQPKDYSLNSVPIYQNILVLNHVKGSHCYTASYHLVAVGCTCVWARSNQT

>O.latipes_NP_001191714.1_IL-17A/F-1

MFSATSFCKEMGRGQKAPLALMMMMMMRMMVTEAAAVPKASKTVPLLLDPSALVPTRIIRPLQNVSISPWTYNTSSDSSLLMPALSEARCLLRGCLNLEGKEDLSLESRPIMHQVLVLRRVRAAGHSYDYHLESRLIAVGCTCVKPVVQVQQ

>O.latipes_NP_001191716.1_IL-17D

MTAGSSRICVLLLLLLLHLAALLDASRVRKKASRTRSCLDLPEEILEQMFGRLSVGVMSAFHHALQLKPQDKLNLSCPSTAGTLTASRTRLPVNLLSVSPWAYRSISYDPTRYPRSIPEAYCLCKGCLIGPSGEESRQYRSTPVYSPSVILRRTGSCAGGRHSYSEIYVSVAVGCTCVPLLEKDREPQSRNQTLQRGAPKARRVSAAGHKG

>O.latipes_NP_001191723.1_IL-17C

MRLNQILIGTVCLVALCACKKCVSVDDLKKIEEKAMRKHGIGIWQHSISSPSSDATGCPVDLYQNWDSKDEKRSVSPWKYTEVTREGYFPPSYMEAQCLCKGCILKEGNNVIESHNFNSKPLVVSRMFLRRVLCEGGNNGTAKTYKLERVQLDVAVGCICVRTRS

>O.latipes_NP_001191717.1_IL-17N

MEQLQLLQVLLLCLLGLQRGAATPLSTQCVEESFCTFNLQDVHGQLVNLPSHVNERSIPTWSYVENIDLNRVPQVIHEASCHSSHACPGLDSGYSLETVPVSLRMPVLKKNPACFSTTGYSVDYELITVACLCVISRHN

>T.rubripes_BAI82582.2_interleukin-17C-2

MTISLGSLLLFGERPLCAGGSQCVSEDEANQRLERFHKNRPTPTFTDHQTTCEKVRQLFEDVAPQLKGEHMRQRSVSPWRYRIDHNINRSPADIAMAECLCKGCILEQKETHDYNSVSVTTHIKVLYKNPCPDDPGKYLMKHEYFKVSVGCICVEPKRSS

>T.rubripes_BAI82581.2_interleukin-17C-1

MKQFLTFVLLLVSACTGESNRCYDEHELSEAANRKLRSHYPQPAEPSPAAAADSSYTCPLELYLQKDLPPHLSGRTVPLGDSFPSSYTEAQCLCSGCILVPDSPQNQVLLTETHDYNSVPIKQNRVFLRKELCADGKKHHLKPVTIQVAVGCTCVRPKTTS

>T.rubripes_BAI82580.1_interleukin-17A/F-3

MQVVSGTLLLLGLLALLHAARNVKSRQQSRPKGRRLRLEIDPSVWPELSSMTAPSLANRSLSPWTYTGSSEESRFPRWIYSAQCLTASCLSLRGEGEDAALEAAPIYYPTLVLHRVPKQRKANKKKGRSSREKYEFQLRTAVVSVGCTCVRPTVIPQQ

>T.rubripes_BAI82579.1_interleukin-17A/F-2

MVLRSCSVVALLISCSALWGFSHSSRPKPPPPSPKCDAMVAFSSQTSSLSEGAGAIHSRSLSPWRWRSTTVKNRIPTTLWEAECTSRFSSGPRLGQPEVHNLNSVPIYQNILVLTRQNNSHCYTASFQLVAVGCTSVRATISHS

>T.rubripes_BAI82578.1_interleukin-17A/F-1

MGHSKATMTVGMLAVMMMVAALAAALPRPGGHLKRSVKANKKSPAVMETVPLQLDPKNLVVTQNIRPLENVSISPWTYNISRDASLFPPLAEARCLFRGCLDSEGQEDQSLESKPIMRQVLLLRKVSSEEGAGHSYHFRLESRLVAVGCTCIRPVVLHHQ

>T.rubripes_BAI82584.1_interleukin-17N

MEQLQLVRVFLLCALALHGCVAMPFTDHCVDESFCTYSLQDYHTQLVELPSHINQRSIASWTYVENIDLNRVPQVIHEASCHTRHSCNGLENTFGLETIPVSLRMPVLKKNPNCFPTSSYSLDFELITIACICAFSRNS

>G.gallus_XP_003641993.2_interleukin-17C

MGWLGALALLSAVVLCRCLRRPAVPTHHHPHVRCYSSGELRDGEAPAHLLGRSLRWEQHVPVQLVPQIEAAQRRRRRREHSCPALQLRAGLRSEPHERSISPWRYRIDEDEDRYPRKLAFAECLCTGCVDVKTGRETTALNSVPIHQTMMVLRRKPCPRPSSPGLITFDVDYIRVPVGCTCVLPRTGR

>G.gallus_XP_426223.4_interleukin-17F

MAFASCAAVFRSLLLVLVLTLTVWSSPHGKVVRPRPRKDGGSEKLSEDCLNQKDPSFPTMVKVDIRIGSSDPASRMIHDIRNRSLAPWNYRLDEDPNRFPQVIADAECRLLGCLNSLGQEDRSLNSVPITQEILVLRREQRGCQPTYHLEKKLITVGCTCAAPVIQHQS

>G.gallus_NP_989791.1_interleukin-17F precursor

MSPIPYSPLFRPLLLVLLAMLSASISAHGKVIRPGLEPESLFKKADAGCLTQKDGKFPQTVRVNISISNMNQDTKVTLDISKRSLAPWDYRIDEDHNRFPRLVADAQCRHSRCVNSAGQLDHSVNSVPIKQEILVLRREPKGCQHSYRLEKKMITVGCTCVTPLIQHQA

>G.gallus_XP_004944893.1_interleukin-17B isoform X4

MERAPNLLLLCIFTFAMVLVPEAKDESRAAKGRRRGPSRLPTAASALAWAPDDPYTPMEDYERSIQDMVHQLRNGSEPGDTKCQVNLRLWRSNRRSLSPWAYRCSGDHSSSISKQPRCSAPHVAPQWYCPAPSPPRCPHPTCHSHRPPNLPLLSLSSSINHDATRIPADIPEARCLCTGCINPFTMQEDRTMASIPIYSRLPVRRLLCQAPGKVGHKASGRKKCHKKYQTVMETIAVGCTCIF

>X.tropicalis_NP_001107719.1_interleukin-17D precursor

MMLLFGILIASCNGSKPVKRPPPKPKSCADRPEEHLEQVYGRLAAGMLSAYHHTLQLQPLDKENISCPAGTQGRGAGDGKQRLPVNIHSISPWAYRISYNPTRYPKYIPEAYCLCKGCLTGLLGEEDLNFRSMPVYMPTVILRRTTSCAGGRYVYEEEYITIPVGCTCVPEQEKGAELLESVNSSLEKEKFKLPLNKSEKPLAN

>X.tropicalis_XP_004915038.1_interleukin-17A-like

MSEQQASMLLVTLVLFMAGSAFSKKCPLTKEMFSFPQNIKLSLNFSSTGQENPRSGETHMRSISPWNYSINMDKNRFPSVINEAVCVHNGCLDAEGNVDISLRSAPIQQTILVLRREVRGCSTSFWLEKQTVTVGCTCIRKEILLRPHSMEPNPTGSLYS

>X.tropicalis_XP_002942041.2_interleukin-17C

MNNLCATWLLGLFLCVSMLITGASTRKHPHRHHQHPFCFTNDELEVSFKDIVHQAAGKNMYWDHYQAVHLVKTLEETESRRKRKRRGEPENGCPNLKASTPSGNGRSDLGQRSISPWSYRIDFDENRYPQKLAFAQCLCKGCINAETGKDDASLNSVSVEQTMLVLRKTRCSENSSRYMFELDYIKVPVACTCVIPRN

>X.tropicalis_XP_002932904.1_interleukin-17D-like

MKLLLSLLLALTLASCQGNQIKCKDPSEEYLKNKLFRHAPDAQILLFKPDISPDQELRTCPKSVNHSSTLIQERSISPWSYRINENVNRYPKQILEAYCLCKGCISSHNKGQTTVVSVPFDKEVPVLHKTPKCKKGRFVYKLRFIRIAQLCICRFH

>X.tropicalis_XP_004915036.1_interleukin-17F

MTMGKSYSGMNTYTLAYRGEIKVGYKQEKGKKVLFDCHVTLLLFILGVSTTLSVHGLDLHHLSKGCPPPRGLNLNQPIKVTLNISGQSQALGKDISKRSLAPWDYSFDMDNNRFPSMIAEAKCRYAHCLDAEGNLDLDVNTVPIKQEILVLRREMKGCTPSFKLEKKMVTVGCTCVRPEVKEQQQ

>X.tropicalis_NP_001006699.1_interleukin-17B precursor

MFGSHRLFLLIATSLLVAHALSSDTPNPSKGRKKGHLKGKNTHGTAQDRAKGKASPDPVLGGNSFAPSQLYSLVEDYEQSLMEMVNQLRNNSDVSANRCEVSLRLWLSNRRSLSPWAYSINHDENRIPIDIPEARCLCTGCVNPFTMKEDFSMTSIPIYSKIPVRRRLCEGSSSPIRARRRKKCHKEYMAVMENIAVGCTCIF

>X.tropicalis_XP_004915037.1_interleukin-17A-like

MPNVWMPKVSITLLGINTMTSAHPHVPQGDCQLPADASFPAVVRASIRSGGQIQSLRMEMRNRSLSPWDYVNDQDNNRYPNIIYEAKCRHAGCLDSQGNMDARVNSIPIRQEILVLRREMIGCTPSFRLEKKMVTVGCTCARPIVQHLI

>H.sapiens_NP_612141.1_interleukin-17D precursor

MLVAGFLLALPPSWAAGAPRAGRRPARPRGCADRPEELLEQLYGRLAAGVLSAFHHTLQLGPREQARNASCPAGGRPADRRFRPPTNLRSVSPWAYRISYDPARYPRYLPEAYCLCRGCLTGLFGEEDVRFRSAPVYMPTVVLRRTPACAGGRSVYTEAYVTIPVGCTCVPEPEKDADSINSSIDKQGAKLLLGPNDAPAGP

>H.sapiens_NP_443104.1_interleukin-17F precursor

MTVKTLHGPAMVKYLLLSILGLAFLSEAAARKIPKVGHTFFQKPESCPPVPGGSMKLDIGIINENQRVSMSRNIESRSTSPWNYTVTWDPNRYPSEVVQAQCRNLGCINAQGKEDISMNSVPIQQETLVVRRKHQGCSVSFQLEKVLVTVGCTCVTPVIHHVQ

>H.sapiens_NP_055258.1_interleukin-17B precursor

MDWPHNLLFLLTISIFLGLGQPRSPKSKRKGQGRPGPLAPGPHQVPLDLVSRMKPYARMEEYERNIEEMVAQLRNSSELAQRKCEVNLQLWMSNKRSLSPWGYSINHDPSRIPVDLPEARCLCLGCVNPFTMQEDRSMVSVPVFSQVPVRRRLCPPPPRTGPCRQRAVMETIAVGCTCIF

>H.sapiens_NP_002181.1_interleukin-17A precursor

MTPGKTSLVSLLLLLSLEAIVKAGITIPRNPGCPNSEDKNFPRTVMVNLNIHNRNTNTNPKRSSDYYNRSTSPWNLHRNEDPERYPSVIWEAKCRHLGCINADGNVDYHMNSVPIQQEILVLRREPPHCPNSFRLEKILVSVGCTCVTPIVHHVA

>H.sapiens_NP_037410.1_interleukin-17C precursor

MTLLPGLLFLTWLHTCLAHHDPSLRGHPHSHGTPHCYSAEELPLGQAPPHLLARGAKWGQALPVALVSSLEAASHRGRHERPSATTQCPVLRPEEVLEADTHQRSISPWRYRVDTDEDRYPQKLAFAECLCRGCIDARTGRETAALNSVRLLQSLLVLRRRPCSRDGSGLPTPGAFAFHTEFIHVPVGCTCVLPRSV

>H.sapiens_NP_073626.1_interleukin-25 isoform 1 precursor

MRERPRLGEDSSLISLFLQVVAFLAMVMGTHTYSHWPSCCPSKGQDTSEELLRWSTVPVPPLEPARPNRHPESCRASEDGPLNSRAISPWRYELDRDLNRLPQDLYHARCLCPHCVSLQTGSHMDPRGNSELLYHNQTVFYRRPCHGEKGTHKGYCLERRLYRVSLACVCVRPRVMG

2) Some IL17 protein sequences that contain incomplete IL-17 domains

>C.elegans_CDH93392.1 Protein C44B12.6 isoform a

MHEAFSRLINRRDEDMFSFFQPDSQIFLEYQPLHRHMLTPKSRDCSEPSIDKSSEVLPDQPLSERSICPYHHILNYDEKRIPAAISEVECSCPHVKVHGGIIHCEPMMYNMRVMLFDDSCDKYVERVQKVALACVPVFSNHISSGTHSHSLPTPPSTPL

>P.fucata_pfu_aug1.0_8548.1_09780.t1

MNLTHPDPLEYLLLPDSNQNKNYIPAPVERPSFINGIKDCPNSQTNLNVTVTTPLSERATCPFYFVSTFDARRYPQHITEARCSCSHCLEYHGLSSRTRCEPVYRDVKLLVKTECHNSVWQYSVGIYKMQESCTCAVPRQIRNQQTGNKISNSGEPIPM

>S.purpuratus_SPU_030197.1

MASTMKVQRFITICLIYAPLLSAFVVKYEDMAIESIISQTEKSEADRLPEVFSKNNTESSMASLVTENIANIERHEAHQPIKTANGDVHTALKRSACREPSGADLEVQLQLFRSGFVTTEEPTGAFGAGVDSLESIRDSGFPVYWEGEDCRTAPSDLWTIFPTTSLNGRTACPWRLVQNSKPNRYPRDIQYAQCACTKCAEIPLDSFSIANSCRPVLRDEHVLNRTDQCVDGEYVSNGTQIISPPHTAFSMGYVYSRGFKIMNCFRCKIYLNAINPLLTGTGGRRVKKGDMEVIKNGMGY
