# Supplementary figures and images for: Comparative and Evolutionary Analysis of the Interleukin 17 Gene Family in Invertebrates
Source: PLoS One. 2015 Jul 28;10(7):e0132802. doi: 10.1371/journal.pone.0132802 (PMC4517768; doi:10.1371/journal.pone.0132802)

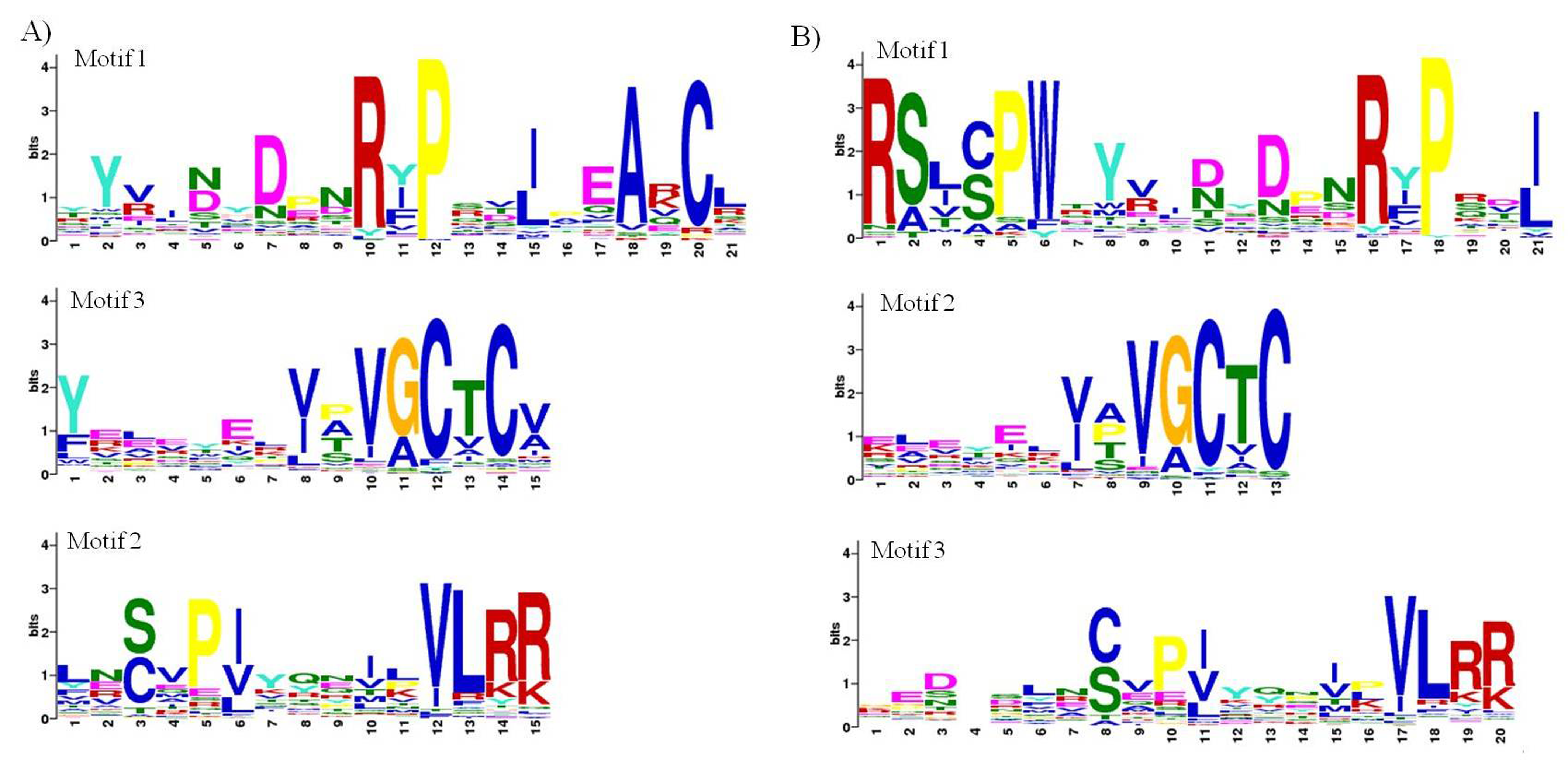

Supplement: S1 Fig — The motifs were analyzed by MEME 4.9.1. The full-length IL-17 proteins and IL-17 domains have similar sequence logos for the motifs, but the motifs have different names. (TIF) [file pone.0132802.s002.tif]

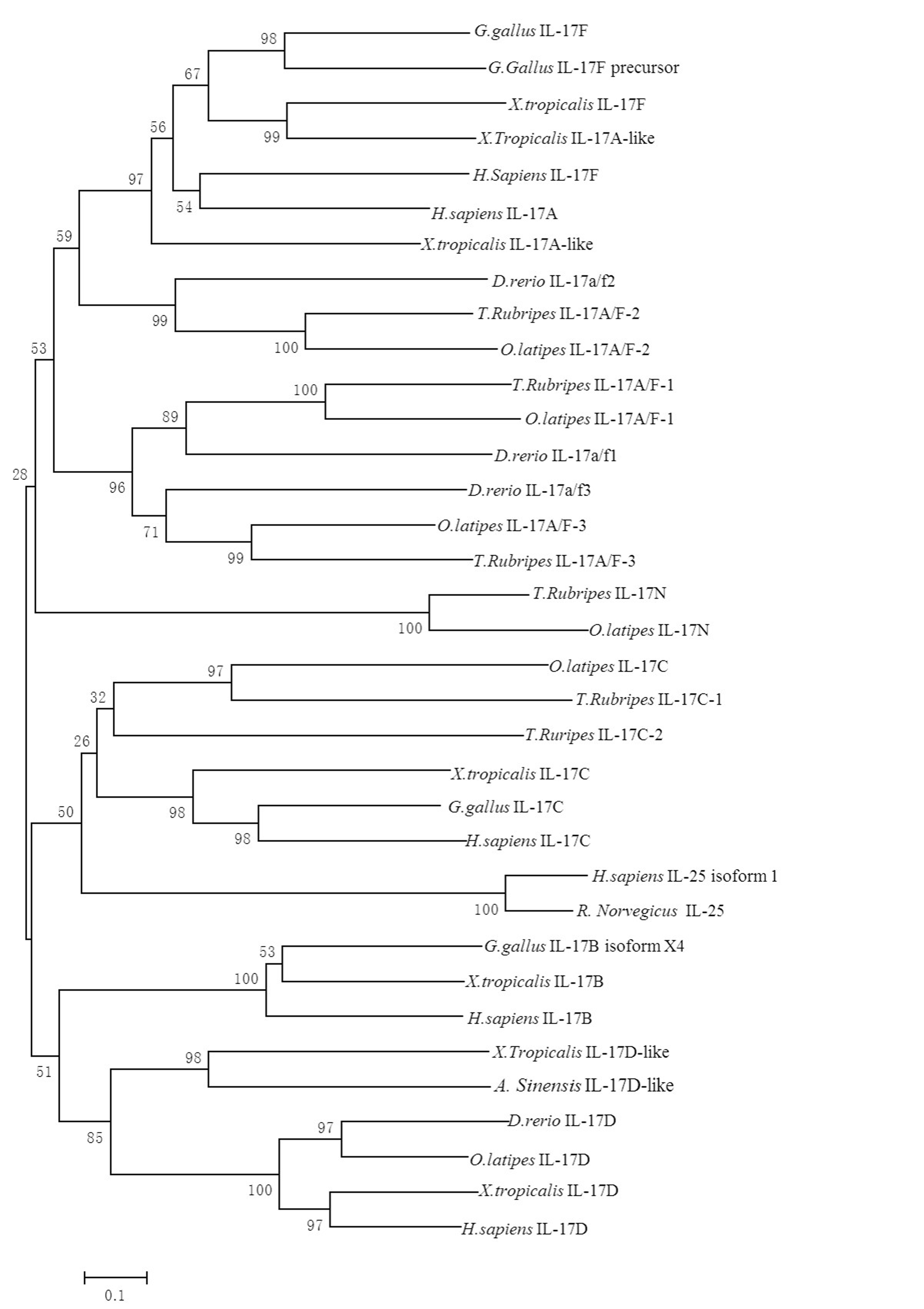

Supplement: S2 Fig — The extra IL-17 protein sequences (Rattus norvegicus IL-25 (NP_001178936.1), Alligator sinensis IL-17D-like isoform X2 (XP_006022303.1)) have not been listed in S1 Dataset. (TIF) [file pone.0132802.s003.tif]
